# Demographic and Socioeconomic Factors Associated with Fungal Infection Risk, United States, 2019 

**DOI:** 10.3201/eid2810.220391

**Published:** 2022-10

**Authors:** Emily Rayens, Mary Kay Rayens, Karen A. Norris

**Affiliations:** University of Georgia, Athens, Georgia, USA (E. Rayens, K.A. Norris);; University of Kentucky, Lexington, Kentucky, USA (M.K. Rayens)

**Keywords:** Fungi, mycoses, socioeconomic factors, health care costs, social class, minority health, healthcare disparities, United States

## Abstract

Fungal infections cause substantial rates of illness and death. Interest in the association between demographic factors and fungal infections is increasing. We analyzed 2019 US hospital discharge data to assess factors associated with fungal infection diagnosis, including race and ethnicity and socioeconomic status. We found male patients were 1.5–3.5 times more likely to have invasive fungal infections diagnosed than were female patients. Compared with hospitalizations of non-Hispanic White patients, Black, Hispanic, and Native American patients had 1.4–5.9 times the rates of cryptococcosis, pneumocystosis, and coccidioidomycosis. Hospitalizations associated with lower-income areas had increased rates of all fungal infections, except aspergillosis. Compared with younger patients, fungal infection diagnosis rates, particularly for candidiasis, were elevated among persons >65 years of age. Our findings suggest that differences in fungal infection diagnostic rates are associated with demographic and socioeconomic factors and highlight an ongoing need for increased physician evaluation of risk for fungal infections.

## Abstract 2

Diagnosis disproportionately affected minority and low-income populations, underscoring the need for broad public health interventions.

Fungal pathogens cause millions of deaths and tens of millions of infections globally every year (*1*). Fungal infections are primarily opportunistic, causing moderate to severe disease in immunocompromised patients. Fungal infections also are associated with increased illness rates and substantial healthcare costs, resulting in $6.7 billion in hospitalization costs in the United States in 2018 (*2*). In addition, fungal infections doubled the average length and cost of hospital stays and risk for death among patients with >1 associated risk condition (*2*). Despite the considerable medical and economic burden of fungal infections, standardized diagnostic and treatment guidelines are lacking.

The risk for serious fungal infection continues to move away from HIV-associated infections (*3*), and increasingly affect patients with certain underlying conditions, including chronic obstructive pulmonary disease (COPD) (*4*), cirrhosis (*5*), cystic fibrosis (*6*), diabetes (*7*,*8*), influenza (*9*,*10*), and tuberculosis (*11*). Increased infection rates also have been reported among persons being treated for asthma (*12*,*13*), autoimmune disorders (*14*,*15*), and cancer (*16*), and among transplant recipients (*17*). 

Interest in the effects of race and ethnicity and socioeconomic status on fungal infections and associated patient outcomes has increased (*18*,*19*), especially because diagnosed fungal infections have increased since 2010 (*3*). Previous studies documented the relationship between health disparities and fungal infections (*18*,*19*), but not as a main analytic focus, and studies across multiple fungal pathogens are lacking. We describe diagnosed fungal infections and associated risk conditions by key demographic variables, including race and ethnicity and socioeconomic status.

## Methods

### Data Sources

We used hospital discharge data from the National Inpatient Sample (NIS), Healthcare Cost and Utilization Project (HCUP), from the Agency for Healthcare Research and Quality (*20*). NIS is the largest database of US hospitalization data, covering >96% of the population (*20*). HCUP data comprise hospitalizations, rather than unique patients. We use the term patient to refer to inpatient status; we acknowledge that a specific patient might be included >1 time in our analyses. For total population per income quartile, we used 2006–2010 American Community Survey (*21*) results to estimate population percentages, then adjusted these to the 2019 population.

### Element Descriptions

We used codes from the International Classification of Diseases, 10th Revision (ICD-10), to identify at-risk patients and invasive and noninvasive fungal infections, as previously described (*2*) (Table). We defined fungal infections and associated risk conditions when relevant ICD-10 codes were recorded as any diagnosis in the hospitalization record. Sex, race, and ethnicity data were provided by patient records in NIS. HCUP excludes the data for sex when the state level patient record identifies the patient as both nonfemale and nonmale. Ethnicity took precedence over race in the HCUP database when both were provided as distinct values in the patient record.

**Table:** Number of risk conditions and fungal infections diagnosed among hospitalized patients, United States, 2019*

Fungal infections and risk conditions	ICD-10 code	No. cases diagnosed
Fungal infections		
Aspergillosis	B44	17,825	
Invasive	B44.0, B44.1, B44.7	8,875	
Noninvasive	B44.2, B44.8	4,210	
Candidiasis	B37	457,080	
Invasive	B37.1, B37.5, B37.6, B37.7	19,920	
Noninvasive	B37.0, B37.2, B37.3, B37.4, B37.8	396,765	
Coccidioidomycosis	B38	8,990	
Cryptococcosis	B45	4,900	
Histoplasmosis	B39	4,880	
Mucormycosis	B46	1,370	
Pneumocystosis	B59	9,725	
Other	B35, B36, B40–B43, B47, B48	145,925	
Unspecified mycoses	B49	15,540
Risk conditions		
Asthma	J45–J46	2,273,360	
Autoimmune conditions	G35, G70, K90, L93, M05, M35	483,850	
Cancer	C00–C97	2,869,790	
Chronic obstructive pulmonary disease	J44	4,402,564	
Cirrhosis	K74	468,950	
Cystic fibrosis	E84	29,465	
Diabetes mellitus	E10–E14	8,376,979	
End-stage renal disease	D17	32,665	
HIV	B20–B24	109,180	
Immunosuppressive disorders	D80–D89	224,100	
Influenza	J09–J11	276,950	
Myelodysplastic syndrome	D46	82,170	
Neutropenia	D70	194,870	
Osteomyelitis	M86	385,450	
Pneumonia	J12–J18	2,552,504	
Sepsis	A40–A41	2,820,729	
Transplant history	Z94	266,580	
Transplant complications	T86	145,540	
Tuberculosis	A16–A19	3,690

*Data from ICD-10 codes listed in the Healthcare Cost and Utilization Project, 2019 (*20*). ICD-10, International Classification of Diseases, 10th Revision.

The HCUP dataset predefines each annual income quartile (Q) according to estimated median household income in US dollars of residents living within a patient’s postal code. For 2019, Q1 was $1–$47,999, Q2 was $48,000–$60,999, Q3 was $61,000–$81,999, and Q4 was >$82,000. We defined insurance type by the expected primary payer type to which the hospital visit was billed in the HCUP NIS dataset.

We defined age groups as pediatric (0–17 years of age), adult (18–64 years of age), and senior (>65 years of age). We defined urban-rural status, as previously described (*22*), and considered counties with >50,000 inhabitants as urban. We calculated rate ratios (RRs) and 95% CIs by using SAS version 9.4 (SAS Institute Inc., https://www.sas.com). We used Prism software (GraphPad Software Inc., https://www.graphpad.com) to create figures.

## Results

Nearly 60,000 invasive fungal infections were reported during US hospitalizations in 2019, ≈10% of all diagnosed fungal infections among hospitalized patients. Another 391,000 noninvasive infections, primarily dermophyte, also were diagnosed.

### Fungal Infections and Risk Conditions by Sex

**Figure 1:**
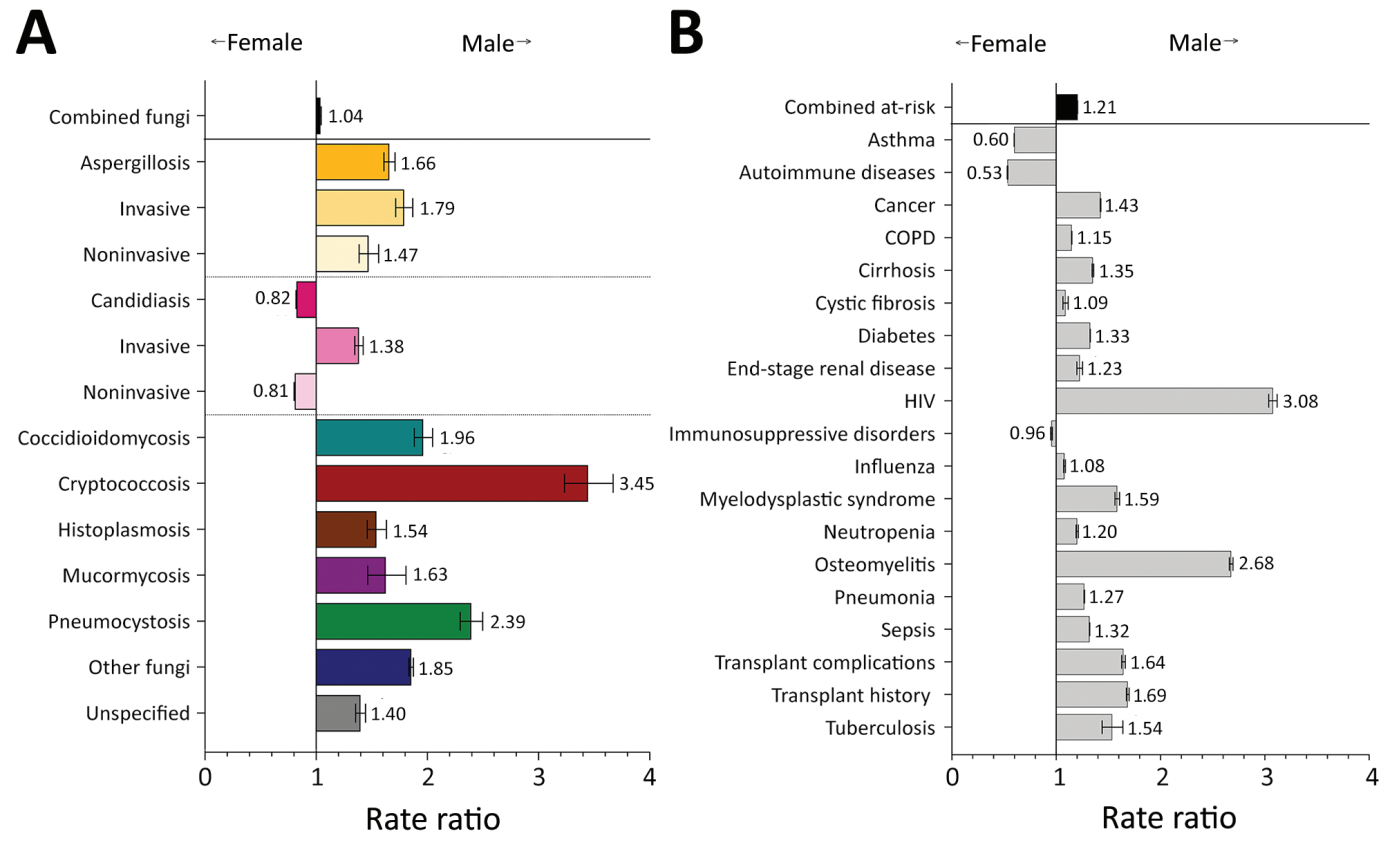
Comparison of rate ratios for fungal infections and risk conditions by sex among hospitalized patients, United States, 2019. A) Diagnosed fungal infections; B) risk conditions. Bars and numerals indicated rate ratios; error bars indicate 95% CIs. COPD, chronic obstructive pulmonary disease.

Invasive fungal infections were diagnosed more frequently in male patients, at 1.4–3.4 times the rate for female patients (Figure 1, panel A; Appendix Table 1). We observed the greatest differences between male and female patients in coccidioidomycosis (RR 2.0, 95% CI 1.9–2.1), pneumocystosis (RR 2.4, 95% CI 2.3–2.5), and cryptococcosis (RR 3.4, 95% CI 3.2–3.7) diagnoses. Noninvasive candidiasis, including vulvovaginal candidiasis, was the only diagnosis made more frequently in female patients, at 1.2 (95% CI 1.2–1.2) times the rate for male patients.

Male patients had >1 fungal-associated risk condition diagnosed at 1.2 (95% CI 1.2–1.2) times the rate for female patients (Figure 1, panel B). Of 19 risk conditions we analyzed, 16 were diagnosed more frequently in male patients. We observed the greatest differences in risk conditions between male and female patients for HIV (RR 3.1, 95% CI 3.0–3.1) and osteomyelitis (RR 2.7, 95% CI 2.7–2.7). Asthma (RR 1.7, 95% CI 1.7–1.7), autoimmune diseases (RR 1.9, 95% CI 1.9–1.9), and immunosuppressive disorders (RR 1.1, 95% CI 1.0–1.1) were diagnosed more frequently in female patients.

### Fungal Infections and Risk Conditions by Race and Ethnicity

Overall, risk conditions and fungal infections were diagnosed among racial and ethnic subgroups at rates generally consistent with the current racial and ethnic composition of the United States; most (65.9%) cases were diagnosed in non-Hispanic White patients. However, we noted deviations that highlight racial and ethnic health disparities.

**Figure 2:**
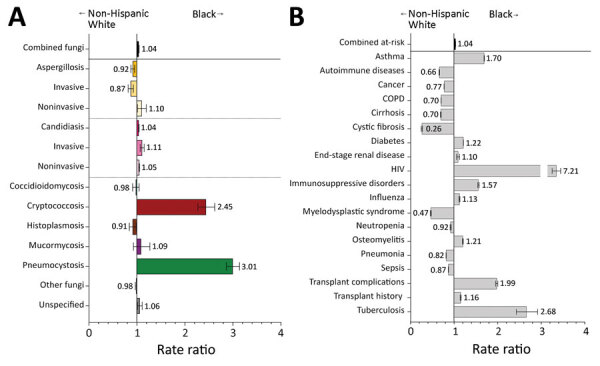
Comparison of rate ratios for fungal infections and risk conditions among non-Hispanic White and Black hospitalized patients, United States, 2019. A) Diagnosed fungal infections; B) risk conditions. Bars and numerals indicated rate ratios; error bars indicate 95% CIs. COPD, chronic obstructive pulmonary disease.

Among Black patients, cryptococcosis was diagnosed at 2.5 (95% CI 2.3–2.6) and pneumocystosis at 3.0 (95% CI 2.9–3.2) times the rates for non-Hispanic White patients (Figure 2, panel A). The transplant history rate appeared similar, but Black patients were twice as likely as non-Hispanic White patients to have transplant complications during hospitalization, particularly for heart and kidney transplants (Figure 2, panel B; Appendix Table 1). HIV was diagnosed among Black patients at 7.2 (95% CI 7.1–7.3) and tuberculosis at 2.7 (95% CI 2.4–2.9) times the rates for non-Hispanic White patients.

**Figure 3:**
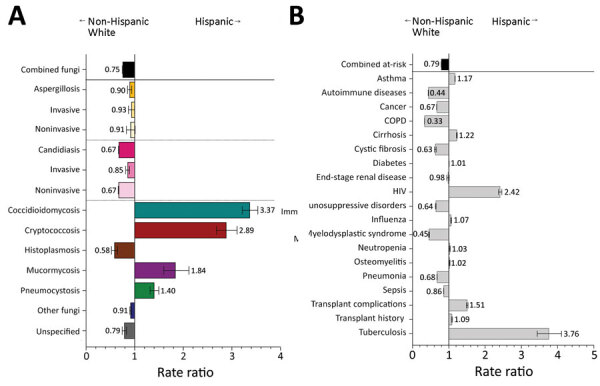
Comparison of rate ratios for fungal infections and risk conditions among hospitalized non-Hispanic White and Hispanic patients, United States, 2019. A) Diagnosed fungal infections; B) risk conditions. Bars and numerals indicated rate ratios; error bars indicate 95% CIs. COPD, chronic obstructive pulmonary disease.

Hispanic patients had fungal infections diagnosed at 0.8 (95% CI 0.8–0.8) times the rate for non-Hispanic White patients; Hispanic patients had decreased rates of aspergillosis, candidiasis, and histoplasmosis (Figure 3, panel A). Rates for coccidioidomycosis (RR 3.4, 95% CI 3.2–3.5), cryptococcosis (RR 2.9, 95% CI 2.7–3.1), and pneumocystosis (RR 1.4, 95% CI 1.3–1.5) were higher among Hispanic than non-Hispanic White patients. HIV was diagnosed among Hispanic patients at 2.4 (95% CI 2.4–2.5) times and tuberculosis at 3.8 (95% CI 3.4–4.1) times the rates for non-Hispanic White patients (Figure 3, panel B). Transplant complications were also moderately elevated in the Hispanic patient cohort.

**Figure 4:**
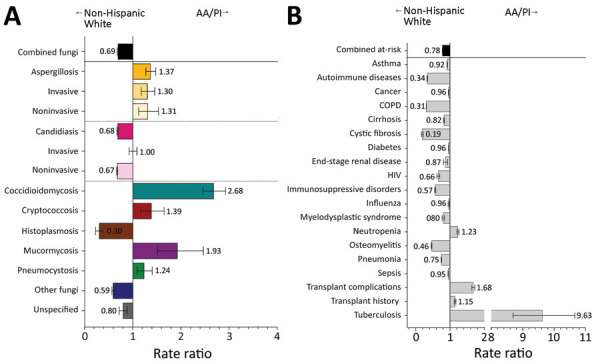
Comparison of rate ratios for fungal infections and risk conditions among hospitalized non-Hispanic White and AA/PI patients, United States, 2019. A) Diagnosed fungal infections; B) risk conditions. Bars and numerals indicated rate ratios; error bars indicate 95% CIs. AA/PI, Asian American/Pacific Islander; COPD, chronic obstructive pulmonary disease.

The overall rate of fungal infection diagnosis in Asian American and Pacific Islander (AA/PI) patients was reduced (RR 0.7, 95% CI 0.7–0.7) compared with non-Hispanic White patients (Figure 4, panel A). Aspergillosis (RR 1.4, 95% CI 1.3–1.5), coccidioidomycosis (RR 2.7, 95% CI 2.5–2.9), and mucormycosis (RR 1.9, 95% CI 1.5–2.5) rates were higher for AA/PI than for non-Hispanic White patients. AA/PI patients had >1 fungal-associated risk condition diagnosed at 0.8 (95% CI 0.8–0.8) times the rate for non-Hispanic White patients (Figure 4, panel B). Transplant complications were moderately elevated in the AA/PI cohort, but tuberculosis diagnoses were 9.6 (95% CI 8.7–10.7) times those for non-Hispanic White patients.

**Figure 5:**
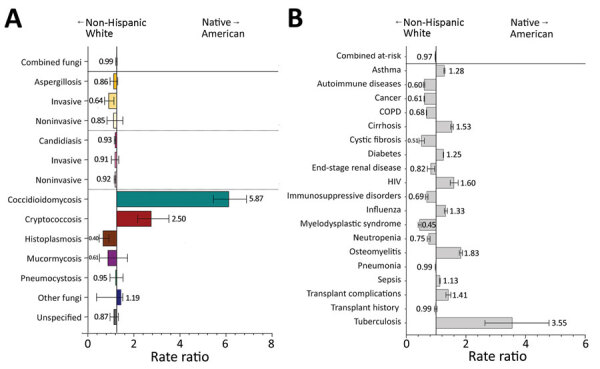
Comparison of rate ratios for fungal infections and risk conditions among hospitalized non-Hispanic White and Native American patients, United States, 2019. A) Diagnosed fungal infections; B) risk conditions. Bars and numerals indicated rate ratios; error bars indicate 95% CIs. COPD, chronic obstructive pulmonary disease.

Native American patients had coccidioidomycosis diagnosed at 5.9 (95% CI 5.2–6.6) times the rate for non-Hispanic White patients (Figure 5, panel A). Native American patients also had higher rates of cryptococcosis (RR 2.5, 95% CI 1.9–3.3) than non-Hispanic White patients, but the rates of pneumocystosis did not differ between these 2 groups (RR 1.0, 95% CI 0.7–1.3). Rates of invasive aspergillosis and histoplasmosis were moderately reduced among Native American patients. For risk conditions, Native American patients had HIV diagnosed at 1.6 (95% CI 1.5–1.7), osteomyelitis at 1.8 (95% CI 1.8–1.9), and tuberculosis at 3.6 (95% CI 2.6–4.8) times the rates for non-Hispanic White patients (Figure 5, panel B).

### Fungal Infections and Risk Conditions by Income

**Figure 6:**
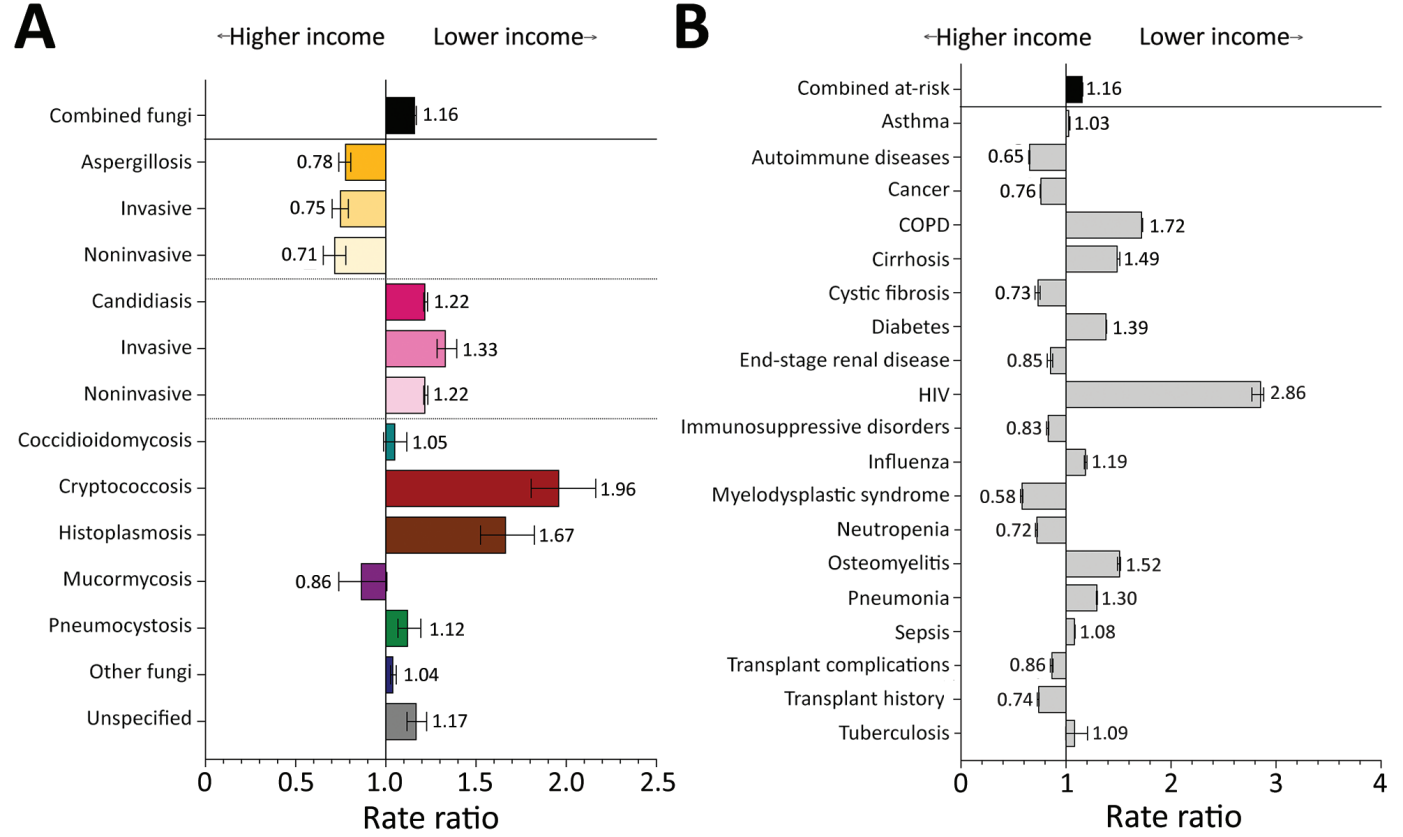
Comparison of rate ratios for fungal infections and risk conditions by income among hospitalized patients, United States, 2019. A) Diagnosed fungal infections; B) risk conditions. Income levels were determined by postal code; patients from postal codes with incomes in the highest quartile were compared with patients from postal codes with incomes in the lowest quartile. Bars and numerals indicated rate ratios; error bars indicate 95% CIs. COPD, chronic obstructive pulmonary disease.

Of 35.5 million hospitalizations in 2019, nearly one third were associated with residence in lower income areas (Appendix Table 2). Patients from Q1 postal codes had 1.6 times the hospitalization rate as patients from Q4 areas. Fungal infections were diagnosed in patients from Q1 postal codes at 1.2 (95% CI 1.2–1.2) times the frequency of patients from Q4 postal codes (Figure 6, panel A; Appendix Table 2). Cryptococcosis was diagnosed at 2.0 (95% CI 1.8–2.1) and histoplasmosis at 1.7 (95% CI 1.5–1.8) times the rate in Q1 patients as in Q4 patients. The only fungal infection diagnosed more frequently in Q4 patients was aspergillosis (RR 1.3, 95% CI 1.2–1.4).

Q1 patients also had >1 fungal-associated risk condition diagnosed at 1.2 (95% CI 1.2–1.2) times the rate for Q4 patients (Figure 6, panel B). COPD, cirrhosis, diabetes, and HIV were diagnosed in Q1 patients at 1.4–2.8 times the rate for Q4 patients. In 2019, Q4 patients were admitted more frequently for conditions associated with higher healthcare costs, including cancer (RR 1.3, 95% CI 1.3–1.3), cystic fibrosis (RR 1.4, 95% CI 1.3–1.4), and organ transplants (RR 1.4, 95% CI 1.3–1.4).

### Fungal Infections and Risk Conditions by Payer Type

**Figure 7:**
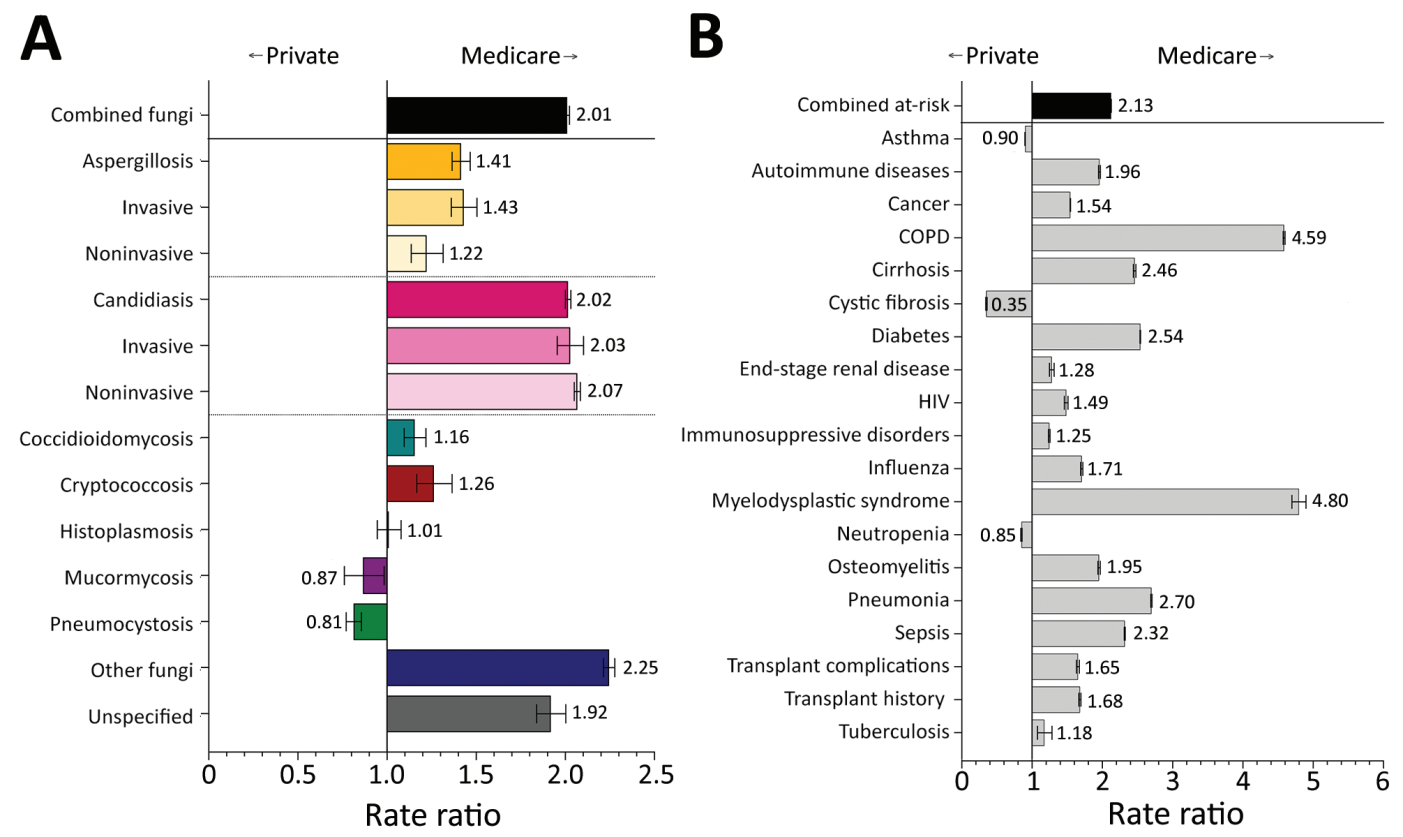
Comparison of rate ratios for fungal infections and risk conditions by billing type (private insurance vs. Medicare) among hospitalized patients, United States, 2019. A) Diagnosed fungal infections; B) risk conditions. Bars and numerals indicated rate ratios; error bars indicate 95% CIs. COPD, chronic obstructive pulmonary disease.

Most (86.7%) persons covered by federally funded Medicare are >65 years of age (*23*). In hospitalizations billed to Medicare, fungal infections were diagnosed at 2.0 (95% CI 2.0–2.0) times the rate for hospitalizations billed to private insurance (Figure 7, panel A; Appendix Table 3). The diagnostic rates for aspergillosis (RR 1.4, 95% CI 1.4–1.5), candidiasis (RR 2.0, 95% CI 2.0–2.0), other fungi (RR 2.2, 95% CI 2.2–2.3), and unspecified fungal infections (RR 1.9, 95% CI 1.8–2.0) were particularly elevated among Medicare patients. Only pneumocystosis (RR 0.8, 95% CI 0.8–0.9) rates were notably lower among hospitalizations billed to Medicare than those billed to private insurance. Hospitalizations billed to Medicare had 2.1 (95% CI 2.1–2.1) times the rate of having >1 fungal-associated risk condition diagnoses as did hospitalizations billed to private insurance (Figure 7, panel B). Rates for 16 of the 19 risk conditions we investigated were elevated in hospitalizations billed to Medicare, and we noted differences in COPD (RR 4.6, 95% CI 2.6–2.6), cirrhosis (RR 2.5, 95% CI 2.4–2.5), diabetes mellitus (RR 2.5, 95% CI 2.5–2.6), myelodysplastic syndrome (RR 4.8, 95% CI 4.7–4.9), pneumonia (RR 2.7, 95% CI 2.7–2.7), and sepsis (RR 2.3, 95% CI 2.3–2.3). Conversely, cystic fibrosis was diagnosed at just over one third the frequency (RR 0.4, 95% CI 0.3–0.4) in hospitalizations billed to Medicare compared with those billed to private insurance.

**Figure 8:**
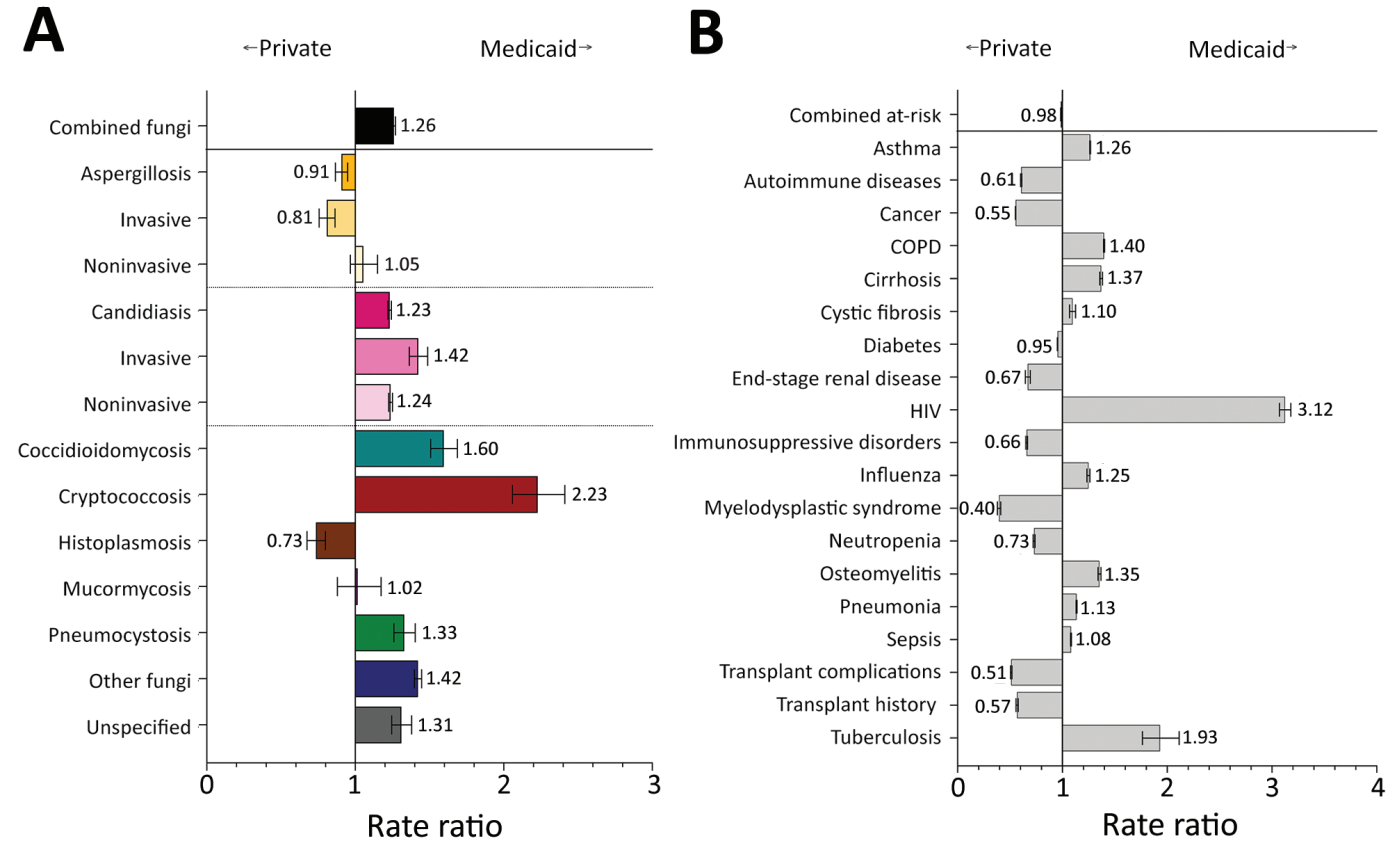
Comparison of rate ratios for fungal infections and risk conditions by billing type (private insurance vs. Medicaid) among hospitalized patients, United States, 2019. A) Diagnosed fungal infections; B) risk conditions. Bars and numerals indicated rate ratios; error bars indicate 95% CIs. COPD, chronic obstructive pulmonary disease.

Federal- and state-funded Medicaid provides free health insurance to persons with low incomes, disabilities, or both (*24*). Fungal infections were more frequent in hospitalizations billed to Medicaid than those billed to private insurance (Figure 8, panel A). In particular, invasive candidiasis (RR 1.4, 95% CI 1.4–1.5), coccidioidomycosis (RR 1.6, 95% CI 1.5–1.7), cryptococcosis (RR 2.2, 95% CI 2.1–2.4), pneumocystosis (RR 1.3, 95% CI 1.3–1.4), other fungi (RR 1.4, 95% CI 1.4–1.5), and unspecified fungal infections (RR 1.3, 95% CI 1.3–1.4) were diagnosed more frequently, and invasive aspergillosis (RR 0.8, 95% CI 0.8–0.9) and histoplasmosis (RR 0.7, 95% CI 0.7–0.8) were diagnosed less frequently for hospitalizations billed to Medicaid compared with those billed to private insurance. The rates of HIV (RR 3.1, 95% CI 3.1–3.2) and tuberculosis (RR 1.9, 95% CI 1.8–2.1) were higher in hospitalizations billed to Medicaid than those billed to private insurance (Figure 8, panel B). Risk conditions with fewer diagnoses billed to Medicaid than to private insurance included autoimmune diseases (RR 0.6, 95% CI 0.6–0.6), cancer (RR 0.6, 95% CI 0.6–0.6), immunosuppressive disorders (RR 0.7, 95% CI 0.7–0.7), myelodysplastic syndrome (RR 0.4, 95% CI 0.4–0.4), and transplant history (RR 0.5, 95% CI 0.5–0.5).

**Figure 9:**
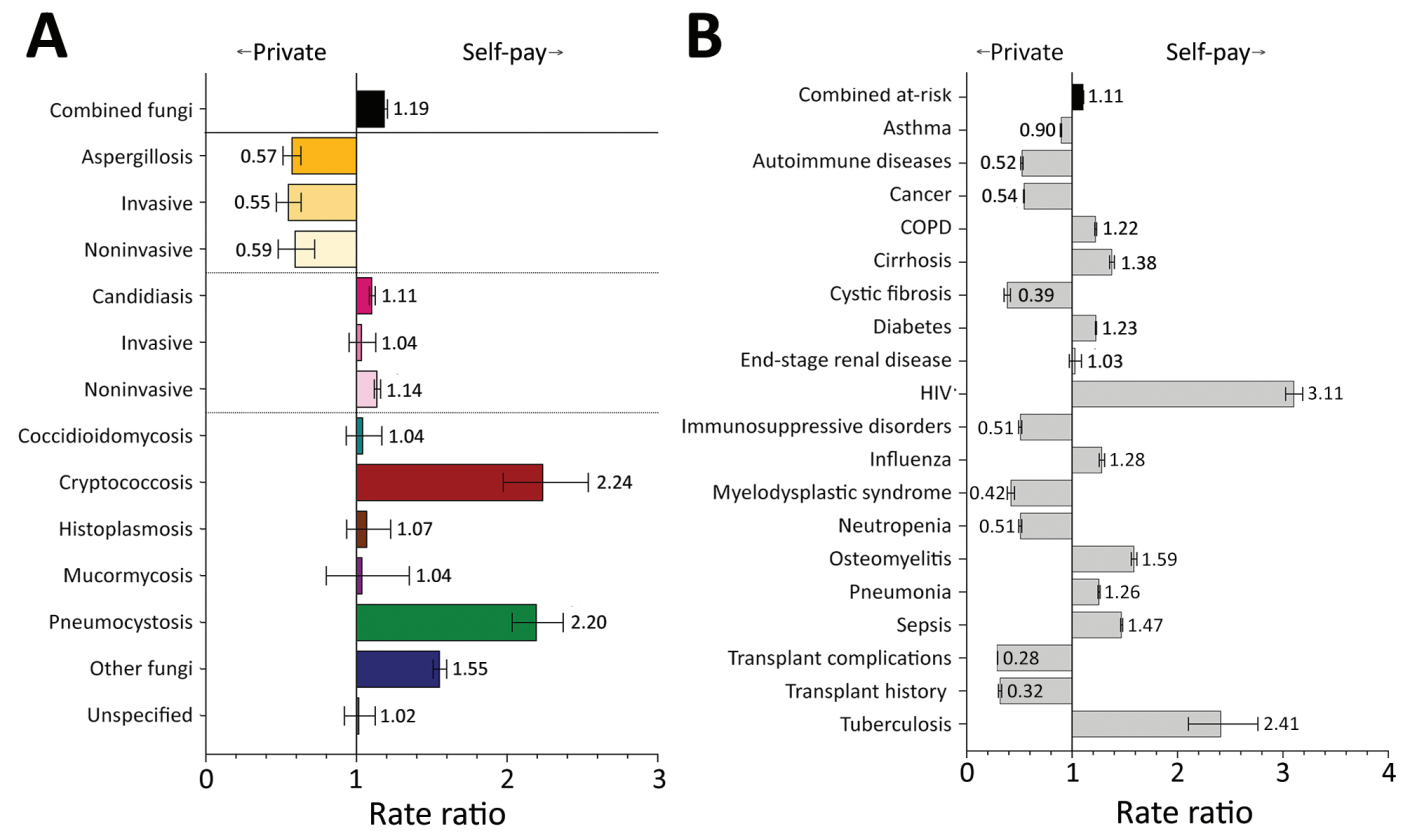
Comparison of rate ratios for fungal infections and risk conditions by billing type (private insurance vs. self-pay) among hospitalized patients, United States, 2019. A) Diagnosed fungal infections; B) risk conditions. Bars and numerals indicated rate ratios; error bars indicate 95% CIs. COPD, chronic obstructive pulmonary disease.

Hospitalizations billed as self-pay represent patients that are uninsured or underinsured (i.e., <30% estimated insurance coverage). These hospitalizations had a lower frequency of diagnoses for aspergillosis (RR 0.6, 95% CI 0.5–0.6) but elevated frequencies for cryptococcosis (RR 2.2, 95% CI 2.0–2.5) and pneumocystosis (RR 2.2, 95% CI 2.0–2.4) (Figure 9, panel A). Rates of HIV (RR 3.1, 95% CI 3.0–3.2) and tuberculosis (RR 2.4, 95% CI 2.1–2.8) were elevated in hospitalizations billed as self-pay compared with private insurance, but other risk conditions were reduced, including autoimmune diseases (RR 0.5, 95% CI 0.5–0.5), cancer (RR 0.5, 95% CI 0.5–0.5), immunosuppressive disorders (RR 0.5, 95% CI 0.5–0.5), myelodysplastic syndrome (RR 0.4, 95% CI 0.4–0.5), and transplant history (RR 0.3, 95% CI 0.3–0.3) (Figure 9, panel B).

### Fungal Infections and Risk Conditions by Age

**Figure 10:**
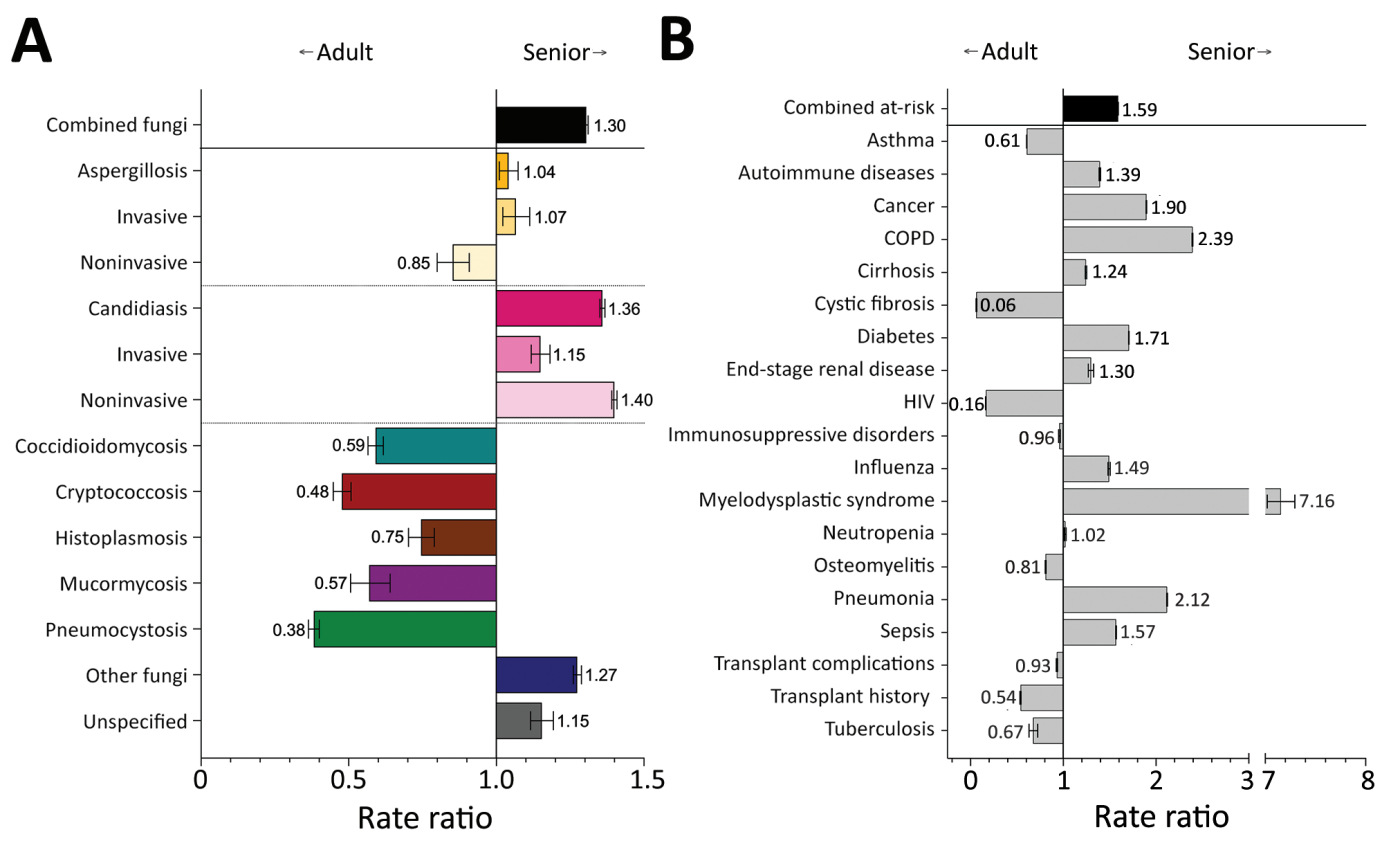
Comparison of rate ratios for fungal infections and risk conditions among adult and senior hospitalized patients, United States, 2019. A) Diagnosed fungal infections; B) risk conditions. Adult patients are persons 18–64 years of age; senior patients are >65 years of age. Bars and numerals indicated rate ratios; error bars indicate 95% CIs. COPD, chronic obstructive pulmonary disease.

Fungal infection diagnosis rates among senior patients were 1.3 (95% CI 1.3–1.3) times that for adult patients. We noted moderate elevation in the rate of invasive aspergillosis diagnoses among senior patients, but noninvasive candidiasis was diagnosed more frequently (RR 1.4, 95% CI 1.4–1.4) among senior than adult patients (Figure 10, panel A; Appendix Table 4). Fungal infections diagnosed less frequently in senior than adult patients included coccidioidomycosis (RR 0.6, 95% CI 0.6–0.6), cryptococcosis (RR 0.5, 95% CI 0.5–0.5), histoplasmosis (RR 0.8, 95% CI 0.7–0.8), mucormycosis (RR 0.6, 95% CI 0.5–0.6), and pneumocystosis (RR 0.4, 95% CI 0.4–0.4). Senior patients had >1 fungal-associated risk condition diagnosed at 1.6 (95% CI 1.6–1.6) times the rate of adult patients (Figure 10, panel B). We also noted elevated rates of COPD (RR 2.4, 95% CI 2.4–2.4), myelodysplastic syndrome (RR 7.2, 95% CI 7.0–7.3), and pneumonia (RR 2.1, 95% CI 2.1–2.1) among senior patients compared with adult patients. Few senior patients had a cystic fibrosis diagnosis, and HIV (RR 0.2, 95% CI 0.2–0.2) diagnoses also were lower than among adult patients.

**Figure 11:**
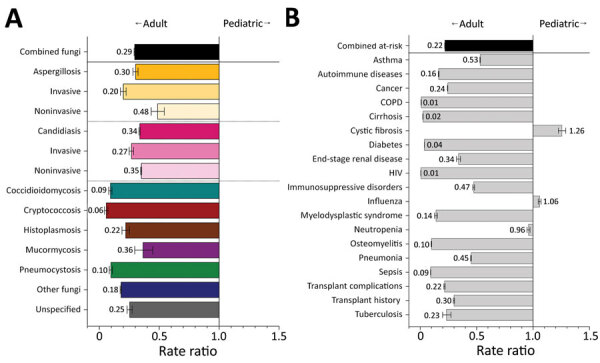
Comparison of rate ratios for fungal infections and risk conditions among adult and pediatric hospitalized patients, United States, 2019. A) Diagnosed fungal infections; B) risk conditions. Adult patients are persons 18–64 years of age; pediatric patients are <17 years of age. Bars and numerals indicated rate ratios; error bars indicate 95% CIs. COPD, chronic obstructive pulmonary disease.

Despite representing 14.9% of hospitalizations in 2019, pediatric patients accounted for only 4.2% of diagnosed fungal infections and had one third the diagnostic rate (RR 0.3, 95% CI 0.3–0.3) of adult patients (Figure 11, panel A); rates of all fungal pathogens and manifestations were reduced. Pediatric patients had >1 fungal-associated risk condition diagnosed at 0.2 (95% CI 0.2–0.2) times the rate for adult patients (Figure 11, panel B). Only the diagnostic rate for cystic fibrosis (RR 1.3, 95% CI 1.2–1.3) was higher among pediatric than adult patients.

### Fungal Infections and Risk Conditions by Rural or Urban Status

**Figure 12:**
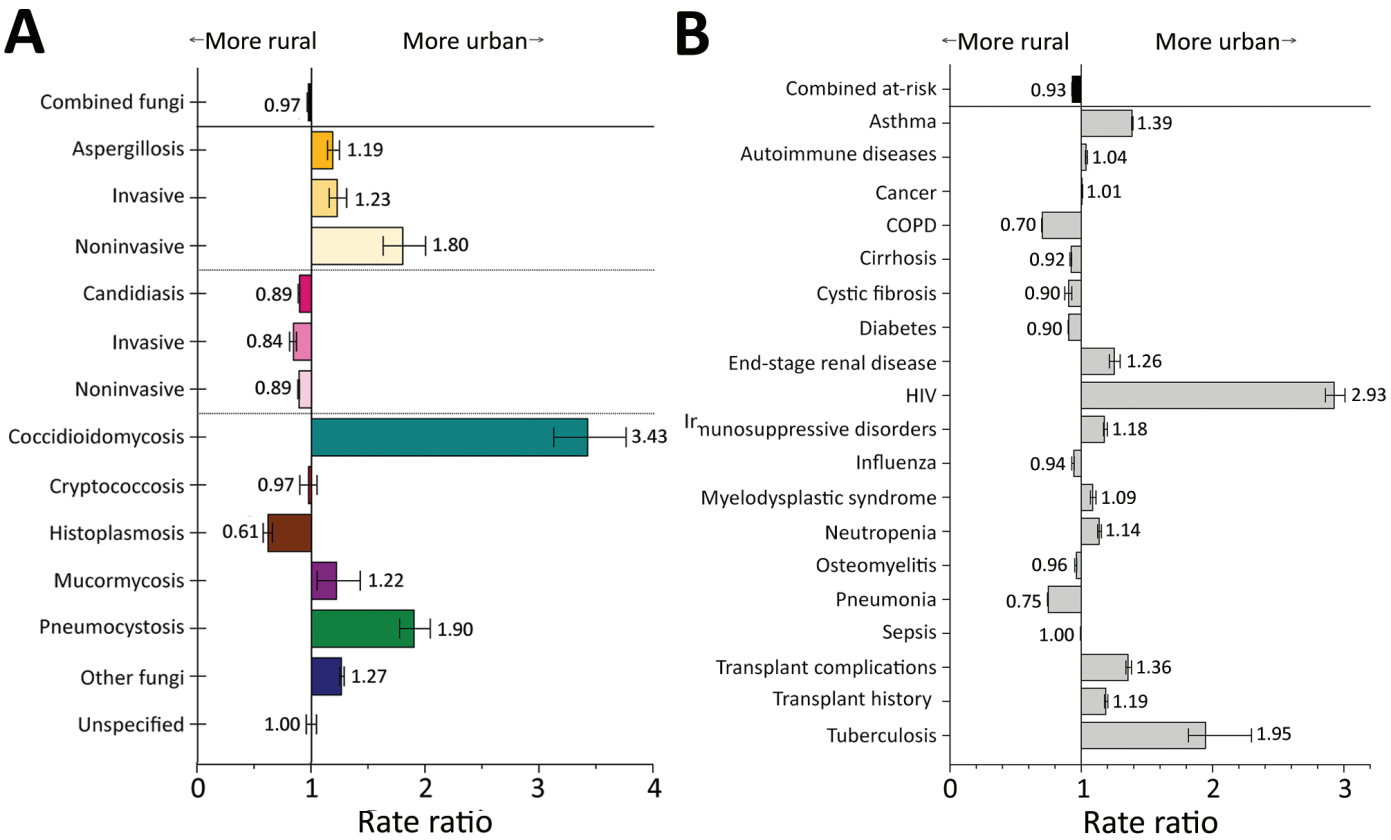
Comparison of rate ratios for fungal infections and risk conditions by residential location (urban vs. rural) among hospitalized patients, United States, 2019. A) Diagnosed fungal infections; B) risk conditions. Persons from more urban settings are considered those whose resident county has a population >50,000. Bars and numerals indicated rate ratios; error bars indicate 95% CIs. COPD, chronic obstructive pulmonary disease.

Among patients from urban areas, certain fungal infections were diagnosed more frequently, including coccidioidomycosis at 3.4 (95% CI 3.1–3.8), pneumocystosis at 1.9 (95% CI 1.8–2.0), and aspergillosis at 1.2 (95% CI 1.1–1.2) times the rates for patients from rural areas (Figure 12, panel A; Appendix Table 5). All aspergillosis infections were diagnosed more frequently in urban patients, but noninvasive aspergillosis (RR 1.8, 95% CI 1.6–2.0) had the greatest difference. Infections diagnosed more frequently among rural patients included candidiasis at 1.1 (95% CI 1.1–1.1) and histoplasmosis at 1.6 (95% CI 1.5–1.7) times the rate for urban patients.

Urban patients had much higher rates of HIV (RR 2.9, 95% CI 2.9–2.9) and tuberculosis (RR 2.0, 95% CI 1.8–2.3) than rural patients (Figure 12, panel B). Asthma (RR 1.4, 95% CI 1.4–1.4) and transplants (RR 1.2, 95% CI 1.2–1.2) also were more common among urban patients, consistent with previous reports (*25*). COPD (RR 1.4, 95% CI 1.4–1.4) and pneumonia (RR 1.3, 95% CI 1.3–1.4) were more frequent among rural patients.

## Discussion

We analyzed rates of fungal infection diagnoses in hospitalizations on the basis of racial and ethnic background and socioeconomic status. Our findings demonstrate that health disparities between racial, ethnic, and socioeconomic groups extend to fungal infections, especially for predisposing risk conditions.

In HCUP NIS, male patients had 1.4–3.5 times the rate of invasive fungal infection diagnoses as female patients, a finding supported by existing literature (*26*). The influence of genetic components by sex has been postulated, as have higher environmental exposure and behavioral risks (*26*,*27*). The relationship between sex and susceptibility is more complex than our analyses can capture, but >1 risk condition for fungal infection was more frequently diagnosed among male patients.

Aspergillosis was diagnosed more frequently in non-Hispanic White and AA/PI patients than in other racial and ethnic groups. As previously described (*3*), invasive aspergillosis is closely associated with stem cell and solid organ transplantation, and noninvasive manifestations, including allergic bronchopulmonary aspergillosis and chronic pulmonary aspergillosis, are more often diagnosed in cystic fibrosis and tuberculosis patients; AA/PI patients have >9 times the rate of tuberculosis diagnoses as non-Hispanic White patients (*28*). In addition, aspergillosis is the only fungal infection diagnosed more frequently in patients from higher income areas. Higher income is associated with higher probability of receiving a transplant (*29*,*30*) and improved patient outcomes in cystic fibrosis care (*31*), possibly because these patients have better access to healthcare facilities and the financial capacity for regular treatment. Aspergillosis likely is more frequently diagnosed in higher income patients because of their ability to continually seek treatment for associated risk factors. Income differences also could relate to cost of living because aspergillosis is more likely to be diagnosed in urban than rural patients (*32*).

Candidiasis was diagnosed more frequently in Black patients. Invasive candidiasis was more frequent in male patients, fitting with previous findings (*33*), but noninvasive candidiasis was more frequent in female patients. Increased rates of candidiasis among senior patients compared with adult patients also is consistent with prior findings (*33*). All candidiasis clinical manifestations were more frequent in patients from lower income areas. Assessments of the relationship of candidiasis and income are lacking, but these diagnoses might be related to the higher frequency of diabetes in patients from low-income areas (*7*). This finding also might be an artifact of the relationship between low income and increased frequency of repeat hospitalizations (*34*). All candidiasis clinical manifestations were diagnosed moderately more frequently in rural patients.

Coccidioidomycosis and histoplasmosis are endemic infections that can affect immunocompetent persons, but severe disease is more common in immunocompromised persons. Coccidioidomycosis is endemic in the US Southwest and histoplasmosis in the Ohio and Mississippi River Valley regions. Our analysis showed coccidioidomycosis was diagnosed more frequently in Hispanic, AA/PI, and Native American adult male patients than in non-Hispanic White or Black, senior, or female patients. Environmental exposure is key in coccidioidomycosis; workers performing soil-disturbing work or exposed to dusty conditions in endemic areas are at increased risk. Black and Hispanic persons are overrepresented in lower wage, more manual labor, and higher risk occupations, including occupations with frequent dust exposure (*35*,*36*). Previous reports noted higher frequencies of coccidioidomycosis in AA/PI and Hispanic male adults residing in urban areas, but older state-level data also indicated increased rates in Black compared with non-Hispanic White male persons (*36*–*38*).

Non-Hispanic White patients had up to 3 times the rate of histoplasmosis as other racial and ethnic groups. Histoplasmosis diagnoses were higher among adult, low-income, and rural patients. These results are supported by previous reports of histoplasmosis predominantly among middle-aged adult White male persons living in rural areas (*39*). These demographic variables likely capture persons with environmental or occupational exposure, including persons employed in construction, agriculture, and forestry industries (*40*).

Historically, cryptococcosis and pneumocystosis were closely tied to HIV, which continues to disproportionately affect Black and Hispanic/Latino populations (*41*). We found cryptococcosis and pneumocystosis were diagnosed in Black and Hispanic patients at 2–3 times the rate for non-Hispanic White patients. HIV, cryptococcosis, and pneumocystosis frequencies also were elevated in Q1 patients and were far more frequent in adult than senior patients, fitting with previous literature (*42*). HIV, cryptococcosis, and pneumocystosis rates were elevated in hospitalizations billed to Medicaid or self-pay and in urban patients.

Incidence of mucormycosis, a rare and often fatal infection, has been rising (*43*). We found mucormycosis diagnoses were more frequent among AA/PI and Hispanic patients than among non-Hispanic White patients. The most common underlying condition for mucormycosis is diabetes mellitus (*43*), but diabetes was not diagnosed more frequently in AA/PI or Hispanic populations in our study. We noted no differences in mucormycosis rates by income or insurance type. Adult patients were more likely to have mucormycosis than senior patients, and we noted a slight elevation in diagnoses among urban patients.

Other fungal infections include primarily superficial cutaneous and mucosal infections, which were diagnosed more frequently in senior patients and in hospitalizations billed to Medicare, consistent with previous studies (*44*). Unspecified mycotic infections also were more frequently diagnosed in senior patients, which could reflect increased mortality and shorter survival times associated with an aging immune response failing to control invasive fungal infections, as previously described (*45*).

Our results are informative, but our data likely underrepresent the true burden of fungal disease in the United States. Evidence suggests that only half of invasive fungal infections are diagnosed before patient death (*46*). The sensitivity and specificity of many ICD-10 codes for fungal infections are unknown, and misclassification is possible. HCUP NIS enabled us to comprehensively study fungal infections; however, unique patients cannot be identified in NIS, so our data likely represent multiple hospitalizations per patient. Data collection also could be a limitation because race and ethnicity analyses are limited by single identifiers and failed to represent patients with multiracial or multiethnic identities. In addition, some previously studied racial and ethnic subgroups might not have been included for this variable in the NIS dataset. Finally, hospitals might have reported a private insurance payer type for patients covered by a Medicare-managed care program administered by a private insurance company, potentially underrepresenting differences between payer types.

In conclusion, we provide a comprehensive summary of fungal infections and associated risk conditions among hospitalized patients, including corresponding rate ratios by demographic and socioeconomic factors. These findings are based on bivariate analysis, but future studies could use a multivariable analysis of the potential predictive weight of demographic and socioeconomic risk factors and >1 comorbidity to measure evaluated risk for fungal infection by type. Our findings suggest that differences in fungal infection diagnostic rates are associated with demographic and socioeconomic factors. Because fungal infections increase mortality rates and healthcare costs, our results highlight an ongoing need for increased physician evaluation of risk for fungal infections, especially among minority and low-income populations that are disproportionately affected.

Appendix Additional information on demographic and socioeconomic factors associated with fungal infection risk, United States, 2019.
