# Supplementary material for: Demographic and Socioeconomic Factors Associated with Fungal Infection Risk, United States, 2019
Source: Emerg Infect Dis. 2022 Oct;28(10):1955–69. doi: 10.3201/eid2810.220391 (PMC9514344; doi:10.3201/eid2810.220391)
Supplement: Appendix — Additional information on demographic and socioeconomic factors associated with fungal infection risk, United States, 2019. [file 22-0391-Techapp-s1.pdf]

Article DOI: https://doi.org/10.3201/eid2810.220391

Demographic and Socioeconomic Factors Associated with Fungal Infection Risk, United States, 2019

Appendix

Appendix Table 1. Demographic characteristics of hospitalized patients with diagnosed fungal infections, United States, 2019*

	Sex		Race or ethnicity							
Infections and risk conditions	M	F	Non-Hispanic White	Black	Hispanic	Asian American/ Pacific Islander	Native American	Other	Unknown	Total
Fungal infections										
Aspergillosis	9,790 (56.8)<br>62.6	7,460 (43.2)<br>37.7	11,240 (65.2)<br>50.5	2,440 (14.1)<br>46.2	1,920 (11.1)<br>45.2	740 (4.3)<br>69.1	105 (0.6)<br>43.4	465 (2.7)<br>39.0	340 (2.0)<br>30.0	17,250 (100)<br>48.7
Invasive	5,115 (58.6)<br>32.69	3,610 (41.4)<br>18.3	5,730 (65.7)<br>25.7	1,185 (13.6)<br>22.5	1,020 (11.7)<br>24.0	360 (4.1)<br>33.6	40 (0.5)<br>16.5	230 (2.6)<br>19.3	160 (1.8)<br>14.1	8,725 (100)<br>24.6
Noninvasive	2,300 (53.8)<br>14.70	1,975 (46.2)<br>10.0	2,695 (63.0)<br>12.1	705 (16.5)<br>13.4	470 (11.0)<br>11.1	170 (4.0)<br>15.9	25 (0.6)<br>10.3	125 (2.9)<br>10.5	85 (2.0)<br>7.5	4,275 (100)<br>12.0
Candidiasis	176,895 (39.4)<br>1,130.5	271,550 (60.5)<br>1,373.4	300,395 (67.0)<br>1,349.5	74,100 (16.5)<br>1,404.2	38,560 (8.6)<br>908.6	9,835 (2.2)<br>917.9	3,025 (0.7)<br>1,250.3	11,460 (2.6)<br>960.3	11,105 (2.5)<br>981.1	448,480 (100)<br>1,266.2
Invasive	10,795 (52.3)<br>68.8	9,825 (47.7)<br>49.7	13,085 (63.6)<br>58.8	3,450 (16.8)<br>65.4	2,130 (10.3)<br>50.2	630 (3.1)<br>58.8	130 (0.6)<br>53.7	640 (3.1)<br>53.6	525 (2.5)<br>53.4	20,590 (100)<br>58.1
Noninvasive	150,740 (39.0)<br>964.8	236,205 (61.0)<br>1,194.7	258,960 (66.9)<br>1,163.4	64,535 (16.7)<br>1,222.9	33,060 (8.5)<br>779.0	8,375 (2.2)<br>781.6	2,590 (0.7)<br>1070.5	9,940 (2.6)<br>833.2	9,520 (2.5)<br>841.0	386,980 (100)<br>1092.6
Coccidioidomycosis	5,655 (60.8)<br>36.1	3,640 (39.2)<br>18.4	4,310 (46.4)<br>19.4	1,000 (10.8)<br>18.9	2,770 (29.8)<br>65.3	555 (6.0)<br>51.8	275 (3.0)<br>113.7	235 (2.5)<br>19.7	150 (1.6)<br>13.3	9,295 (100)<br>26.2
Cryptococcosis	3,545 (73.2)<br>22.7	1,300 (26.8)<br>6.6	2,025 (41.8)<br>9.1	1,175 (24.3)<br>22.3	1,115 (23.0)<br>26.3	135 (2.8)<br>12.6	55 (1.1)<br>22.7	235 (4.9)<br>19.7	105 (2.2)<br>9.3	4,845 (100)<br>13.8
Histoplasmosis	2,710 (55.0)<br>17.3	2,220 (45.0)<br>11.2	3,465 (70.3)<br>15.6	750 (15.2)<br>14.2	385 (7.8)<br>9.1	50 (1.0)<br>4.7	15 (0.3)<br>6.2	100 (2.0)<br>8.4	165 (3.3)<br>14.6	4,930 (100)<br>13.7
Mucormycosis	785 (56.3)<br>5.0	610 (43.7)<br>3.1	755 (54.1)<br>3.4	195 (14.0)<br>3.7	265 (19.0)<br>6.2	70 (5.0)<br>6.5	†	55 (3.9)<br>4.6	50 (3.6)<br>4.4	1,395 (100)<br>3.9
Pneumocystosis	6,225 (65.4)<br>39.8	3,285 (34.5)<br>16.6	4,370 (45.9)<br>19.6	3,115 (32.7)<br>59.0	1,170 (12.3)<br>27.6	260 (2.7)<br>24.3	45 (0.5)<br>18.6	400 (4.2)<br>33.5	155 (1.6)<br>13.7	9,515 (100)<br>26.8
Other	84,405 (59.5)<br>539.5	57,770 (40.5)<br>292.2	93,970 (65.7)<br>422.2	21,930 (15.3)<br>415.6	16,380 (11.5)<br>386.0	2,660 (1.9)<br>248.3	1,210 (0.8)<br>500.1	3,945 (2.8)<br>330.7	2,850 (2.0)<br>251.8	142,945 (100)<br>403.6
Unspecified mycoses	7,685 (52.5)<br>49.1	6,945 (47.5)<br>35.1	9,535 (65.2)<br>42.8	2,405 (16.4)<br>45.6	1,430 (9.8)<br>33.7	365 (2.5)<br>34.1	90 (0.6)<br>37.2	430 (2.9)<br>36.0	375 (2.6)<br>33.1	14,630 (100)<br>41.3
Total hospitalizations‡	280,660 (45.1)<br>1,794.4	341,130 (54.9)<br>1,725.4	411,855 (66.3)<br>1,850.2	101,700 (16.4)<br>1,927.2	59,055 (9.5)<br>1,391.5	13,630 (2.1)<br>12,72.1	4,410 (0.7)<br>1,822.8	16,545 (2.7)<br>1,386.8	14,645 (2.4)<br>1,293.8	621,840 (100)<br>1,755.7
Risk conditions										
Asthma	730,385 (32.1)<br>4,669.9	1,542,620 (67.9)<br>7,801.8	1,277,495 (56.2)<br>5,739.6	515,305 (22.7)<br>9,764.8	285,615 (12.6)<br>6,729.8	56,540 (2.5)<br>5,276.8	17,785 (0.8)<br>7,531.0	70,000 (3.1)<br>5,867.6	50,620 (2.2)<br>4,472.0	2,273,360 (100)<br>6,418.8

	Sex		Race or ethnicity							
Infections and risk conditions	M	F	Non-Hispanic White	Black	Hispanic	Asian American/ Pacific Islander	Native American	Other	Unknown	Total
Autoimmune conditions	143,620 (29.7)	340,180 (70.3)	366,810 (75.3)	57,655 (11.9)	30,575 (6.3)	5,945 (1.2)	2,395 (0.5)	9,920 (2.1)	10,550 (2.2)	483,850 (100)
	918.1	1,720.7	1,648.1	1,092.5	720.42	554.85	989.9	831.5	932.05	1,366.18
Cancer	1,521,080 (53.0)	1,348,585 (47.0)	2,004,325 (69.8)	367,890 (12.8)	254,915 (8.9)	92,485 (3.2)	13,235 (0.5)	76,775 (2.7)	60,165 (2.1)	2,869,790 (100)
	9,724.9	6,821.9	9,002.4	6,971.3	6,006.4	8,631.6	5,470.4	6,435.5	5,315.2	8,101.3
COPD	2,096,330 (47.6)	2,306,059 (52.4)	3,382,299 (76.8)	564,275 (12.8)	214,160 (4.9)	50,165 (1.1)	25,505 (0.6)	78,270 (1.8)	88,390 (2.0)	4,402,564 (100)
	13,398.2	11,660.9	15,192.95	10,692.7	5,046.1	4,681.9	10,335.2	6,560.8	7,808.7	12,428.9
Cirrhosis	242,390 (51.7)	226,530 (48.3)	307,800 (64.6)	50,895 (10.9)	71,760 (15.3)	12,195 (2.6)	5,135 (1.1)	12,650 (2.7)	8,515 (1.8)	468,950 (100)
	1,549.6	1,146.0	1,382.89	964.4	1,690.8	1,138.2	2,122.4	1,060.4	752.2	1,324.1
Cystic fibrosis	13,635 (46.3)	15,825 (53.7)	23,370 (79.3)	1,435 (4.9)	2,805 (9.5)	215 (0.7)	130 (0.4)	670 (2.3)	840 (2.9)	1,465 (100)
	87.2	80.0	105.0	27.2	66.1	20.1	53.73	56.2	74.2	83.18
Diabetes mellitus	4,288,140 (51.2)	4,080,660 (48.8)	5,176,179 (61.8)	1,495,965 (17.9)	993,595 (11.8)	237,985 (2.8)	70,310 (0.8)	229,395 (2.7)	173,550 (2.1)	8,376,979 (100)
	27,421.0	20,681.4	23,257.3	28,327.7	23,411.6	22,211.1	29,061.0	19,228.5	15,332.0	23,653.3
End-stage renal disease	16,085 (49.2)	16,580 (50.8)	20,690 (63.3)	5,390 (16.5)	3,855 (11.8)	865 (2.6)	185 (0.6)	980 (3.0)	700 (2.1)	32,665
	102.8	83.9	92.9	102.1	90.8	80.7	76.5	82.2	61.8	92.22
HIV	77,360 (70.9)	31,780 (29.1)	32,470 (289.7)	61,230 55,470	14,955 (13.7)	1,030 (0.9)	565 (0.5)	3,550 (3.3)	1,140 (1.0)	109,180 (100)
	494.71	160.7	145.9	1,051.1	352.4	96.1	233.5	297.6	100.7	308.2
Immunosuppressive disorders	96,465 (43.1)	127,615 (56.9)	139,775 (62.4)	51,975 (23.2)	17,075 (7.7)	3,825 (1.7)	1,045 (0.5)	5,425 (2.4)	4,980 (2.2)	224,100 (100)
	616.5	645.58	627.93	984.9	402.33	357.0	431.9	454.7	440.0	632.7
Influenza	127,555 (46.1)	149,385 (54.9)	170,445 (61.2)	45,675 (16.5)	34,610 (12.5)	7,915 (2.9)	2,460 (0.9)	8,875 (3.2)	6,970 (2.5)	276,950 (100)
	815.7	755.6	765.7	865.5	815.5	738.7	1,016.8	743.9	615.8	781.9
Myelodysplastic syndrome	45,735 (55.7)	36,435 (44.3)	64,630 (77.4)	7,015 (8.5)	5,505 (6.7)	2,455 (3.0)	310 (0.4)	1,735 (2.1)	1,520 (1.8)	82,170 (100)
	292.3	184.3	285.80	132.93	129.71	229.12	128.13	145.43	134.28	231.97
Neutropenia	94,990 (48.8)	99,845 (51.2)	123,965 (63.6)	27,140 (13.9)	24,295 (12.5)	7,345 (3.8)	1,015 (0.5)	6,495 (3.3)	4,615 (2.4)	194,870 (100)
	607.2	505.0	556.80	514.29	572.45	685.51	419.53	544.43	407.71	550.11
Osteomyelitis	261,815 (67.9)	123,610 (32.1)	241,880 (62.8)	69,260 (18.0)	47,165 (12.2)	5,375 (1.4)	4,810 (1.2)	9,640 (2.5)	7,320 (1.9)	385,450 (100)
	1,673.8	625.1	1,086.52	1,312.44	1,111.32	501.65	1,988.10	808.05	646.68	1,088.18
Pneumonia	1,279,085 (50.1)	1,273,284 (49.9)	1,771,624 (69.4)	344,500 (13.5)	228,525 (9.0)	63,870 (2.5)	19,045 (0.8)	66,150 (2.6)	58,790 (2.3)	2,552,504 (100)
	8,178.8	6,439.4	7,958.2	6,528.1	5,384.6	5,961.0	7,871.8	5,544.9	5,193.7	7,206.2
Sepsis	1,441,205 (51.1)	1,3479,360 (48.9)	1,879,844 (66.6)	388,845 (13.8)	308,215 (10.9)	85,670 (3.0)	23,125 (0.8)	76,960 (2.7)	58,070 (2.1)	2,820,729 (100)
	9,214.2	6,977.3	8,445.0	7,368.4	7,262.3	7,996.6	9,558.2	6,451.0	5,130.1	7,963.8
Transplant history	152,315 (57.1)	114,245 (42.9)	164,120 (61.6)	44,989 (16.9)	34,105 (12.8)	9,055 (3.4)	1,760 (0.7)	7,100 (2.7)	5,545 (2.1)	266,580 (100)
	973.7	577.8	737.2	852.4	801.5	845.1	727.5	595.1	489.9	752.55
Transplant complications	82,290 (56.5)	63,240 (43.5)	74,615 (51.3)	35,235 (24.2)	21,445 (14.7)	6,035 (4.1)	1,145 (0.8)	3,905 (2.7)	3,150 (2.2)	145,540 (100)
	526.1	319.8	335.28	667.7	505.53	563.2	473.3	327.3	278.3	411.0
Tuberculosis	2,025 (54.9)	1,665 (45.1)	1,165 (31.6)	740 (20.5)	835 (22.6)	540 (14.6)	45 (1.2)	270 (7.3)	125 (2.6)	3,390 (100)
	13.0	8.4	5.2	14.0	19.7	50.4	18.6	22.6	8.495	10.4
Total hospitalizations	8,791,520 (48.8)	9,125,959 (51.2)	11,870,000 (65.9)	2,923,465 (16.2)	1,791,595 (10.0)	442,925 (2.5)	125,595 (0.7)	467,400 (2.6)	387,695 (2.2)	18,008,800 (100)
	56,204.9	46,601.8	53,324.4	55,400.5	42,214.4	41,338.1	51,911.6	39,178.7	34,250.3	50,844.2

*Data collected from the Healthcare Cost and Utilization Project (HCUP), 2019 (https://www.hcup-us.ahrq.gov/nisoverview.jsp). Estimates suppressed in keeping with HCUP protocol. Values represent no. (%), population rate/100,000 persons. COPD, chronic obstructive pulmonary disease.
†Estimates suppressed in keeping with HCUP protocol.
‡Totals are summed by pathogen without duplication of values included in invasive/non-invasive types.

Appendix Table 2. Median household income by ZIP code of hospitalized patients with diagnosed fungal infections, United States, 2019*

Population, infections, and risk conditions	Income quartiles				Q4/Q1 population rate (95% CI)
	Q1, <$47,999	Q2, $48,000–$60,999	Q3, $61,000–$81,999	Q4, >$82,000	
Estimated population	816,886,938 (24.97)	766,639,454 (23.43)	816,266,811 (24.95)	872,206,797 (26.66)	NA
Total inpatient visits	10,584,320 (29.88)<br>1,291.35	8,811,375 (24.88)<br>1,145.50	8,494,420 (23.98)<br>1,037.16	6,935,525 (19.58)<br>792.51	0.61 (0.61–0.61)
Fungal infections					
Aspergillosis	4,515 (26.17)<br>42.66	4,170 (24.17)<br>27.33	4,365 (25.30)<br>51.39	3,830 (22.20)<br>55.22	1.29 (1.24–1.35)
Invasive	2,300 (26.36)<br>21.73	2,030 (23.27)<br>23.04	2,180 (24.99)<br>25.66	2,020 (23.15)<br>29.13	1.34 (1.26–1.42)
Noninvasive	1,045 (24.44)<br>9.87	1,035 (24.21)<br>11.75	1,140 (26.67)<br>13.42	960 (22.46)<br>13.84	1.40 (1.28–1.53)
Candidiasis	145,770 (32.50)<br>1,377.23	113,355 (25.28)<br>1,286.46	102,825 (22.93)<br>1,210.50	78,210 (17.44)<br>1,127.67	0.82 (0.81–0.83)
Invasive	6,975 (33.88)<br>65.90	5,100 (24.77)<br>57.88	4,650 (22.58)<br>54.74	3,415 (16.59)<br>49.24	0.75 (0.72–0.78)
Noninvasive	125,970 (32.55)<br>1,190.16	97,925 (25.30)<br>1,111.35	88,380 (22.84)<br>1,040.45	67,535 (17.85)<br>973.75	0.82 (0.81–0.83)
Coccidioidomycosis	2,685 (28.89)<br>25.37	2,250 (24.21)<br>25.54	2,355 (25.34)<br>27.72	1,675 (18.02)<br>24.15	0.95 (0.90–1.01)
Cryptococcosis	1,885 (38.91)<br>17.81	1,175 (24.25)<br>13.34	920 (18.99)<br>10.83	625 (12.90)<br>9.01	0.51 (0.46–0.55)
Histoplasmosis	1,705 (34.58)<br>16.11	1,325 (26.88)<br>15.04	1,125 (22.82)<br>13.24	670 (13.59)<br>9.66	0.60 (0.55–0.66)
Mucormycosis	375 (26.88)<br>3.54	310 (22.22)<br>3.52	415 (29.75)<br>4.89	285 (20.43)<br>4.11	1.16 (0.99–1.34)
Pneumocystosis	3,300 (34.68)<br>31.18	1,975 (20.76)<br>22.41	2,010 (21.12)<br>23.66	1,915 (20.13)<br>27.49	0.89 (0.84–0.94)
Other	43,740 (30.60)<br>413.25	33,870 (23.69)<br>384.39	33,985 (23.77)<br>400.09	27,360 (19.14)<br>394.49	0.96 (0.95–0.97)
Unspecified mycoses	4,915 (33.60)<br>46.44	3,390 (23.17)<br>38.47	3,235 (22.11)<br>38.08	2,750 (18.80)<br>39.65	0.85 (0.82–0.90)
Total hospitalizations†	198,680 (31.95)<br>1,877.12	154,100 (24.78)<br>1,748.88	143,840 (23.13)<br>1,693.35	112,140 (18.03)<br>1,616.89	0.86 (0.86–0.87)
Risk conditions					
Asthma	705,025 (31.03)<br>6,661.04	540,325 (23.75)<br>6,132.11	543,790 (23.88)<br>6,401.72	446,555 (19.62)<br>6,438.67	0.97 (0.96–0.97)
Autoimmune conditions	115,160 (23.80)<br>1,087.94	119,810 (24.60)<br>1,350.98	127,420 (26.33)<br>1,500.04	115,895 (23.95)<br>1,671.03	1.54 (1.52–1.55)
Cancer	758,240 (26.42)<br>7,163.81	693,010 (24.15)<br>7,864.95	716,935 (24.98)<br>8,440.07	656,010 (22.86)<br>9,458.69	1.32 (1.32–1.33)
COPD	1,579,315 (35.87)<br>14,921.27	1,196,794 (27.18)<br>13,582.38	948,400 (21.54)<br>11,164.98	599,800 (13.62)<br>8,648.22	0.58 (0.58–0.58)
Cirrhosis	161,895 (34.52)<br>1,529.92	120,015 (25.59)<br>1,362.05	105,435 (22.48)<br>1,241.23	70,715 (15.08)<br>1,019.61	0.67 (0.66–0.67)
Cystic fibrosis	7,290 (24.74)<br>68.86	7,230 (24.54)<br>82.05	7,960 (27.02)<br>93.71	6,545 (22.21)<br>94.37	1.37 (1.33–1.4)
Diabetes mellitus	2,830,055 (33.78)<br>26,738.19	2,155,979 (25.74)<br>24,468.15	1,911,130 (22.81)<br>22,498.65	1,338,986 (15.98)<br>19,306.19	0.72 (0.72–0.72)

Population, infections, and risk conditions	Income quartiles				Q4/Q1 population rate (95% CI)
	Q1, <$47,999	Q2, $48,000–$60,999	Q3, $61,000–$81,999	Q4, >$82,000	
End-stage renal disease	9,330 (28.6)<br>88.21	7,915 (24.2)<br>89.83	7,645 (23.4)<br>90.00	7,220 (22.1)<br>104.09	1.18 (1.15–1.22)
HIV	51,375 (47.06)<br>485.52	23,385 (21.45)<br>265.40	17,875 (16.37)<br>210.43	11,915 (10.91)<br>171.80	0.35 (0.35–0.36)
Immunosuppressive disorders	62,860 (28.05)<br>593.98	52,260 (23.32)<br>593.10	55,725 (24.87)<br>656.02	49,835 (22.24)<br>718.55	1.21 (1.20–1.22)
Influenza	88,280 (31.88)<br>833.75	70,375 (25.41)<br>798.68	64,935 (23.45)<br>764.44	48,700 (17.58)<br>702.18	0.84 (0.83–0.85)
Myelodysplastic syndrome	19,050 (23.18)<br>179.98	20,055 (24.41)<br>227.60	20,440 (24.88)<br>240.63	21,575 (26.26)<br>311.08	1.73 (1.70–1.76)
Neutropenia	50,455 (25.89)<br>476.70	45,800 (23.50)<br>519.78	49,035 (25.16)<br>577.26	45,995 (23.20)<br>663.18	1.39 (1.37–1.41)
Osteomyelitis	134,405 (34.87)<br>1,269.85	97,805 (25.37)<br>1,109.99	85,910 (22.29)<br>1,011.37	58,515 (15.18)<br>843.70	0.66 (0.66–0.67)
Pneumonia	844,460 (33.08)<br>7,978.41	663,554 (26.00)<br>7,530.66	571,840 (22.40)<br>6,731.95	427,200 (16.74)<br>6,159.59	0.77 (0.77–0.78)
Sepsis	871,895 (30.91)<br>8,237.61	700,770 (24.84)<br>7,952.02	666,555 (23.63)<br>7,846.97	526,720 (18.67)<br>7,594.52	0.92 (0.92–0.93)
Transplant history	68,115 (25.55)<br>643.32	64,850 (24.33)<br>735.98	68,865 (25.83)<br>810.71	60,675 (22.76)<br>874.84	1.36 (135–1.37)
Transplant complications	41,210 (28.32)<br>389.46	34,530 (23.73)<br>391.88	36,410 (25.00)<br>428.63	31,240 (21.46)<br>450.43	1.16 (1.14–1.17)
Tuberculosis	1,155 (31.30)<br>10.92	800 (21.68)<br>9.08	935 (25.34)<br>11.01	690 (18.70)<br>9.95	0.91 (0.83–1.00)
Total hospitalizations	5,710,745 (31.71)<br>53,954.77	4,554,483 (25.29)<br>51,688.70	4,202,835 (23.34)<br>49,477.60	3,226,517 (17.92)<br>46,521.58	0.86 (0.86–0.86)

*Data collected from the Healthcare Cost and Utilization Project, 2019 (https://www.hcup-us.ahrq.gov/nisoverview.jsp). Values represent no. (%), population rate/100,000 persons. COPD, chronic obstructive pulmonary disease; NA, not applicable; Q, quartile.
†Totals are summed by pathogen without duplication of values included in invasive or noninvasive types.
Highlighting indicates the following rate ratios: <0.49 | 0.50–0.75 | 1.25–1.49 | 1.50–1.99 | 2.00–2.99

Appendix Table 3. Payer type of hospitalized patients with diagnosed fungal infections, United States, 2019*

Infections and risk conditions	Payer type						Total
	Medicare	Medicaid	Private	Self-Pay	Other	Unknown	
Fungal infections							
Aspergillosis	8,880 (51.5)	3,105 (18.0)	4.425 (25.7)	375 (2.2)	445 (2.6)	†	17,250 (100)
	61.12	39.17	43.19	24.57	38.93		48.71
Invasive	4,620 (53.0)	1,425 (16.3)	2,280 (26.1)	185 (2.1)	205 (2.3)	†	8,725 (100)
	31.81	17.98	22.24	12.12	17.93		24.63
Noninvasive	1,975 (46.2)	930 (21.8)	1,140 (26.7)	100 (2.3)	125 (2.0)	†	4,275 (100)
	13.59	11.73	11.12	6.55	10.94		33.08
Candidiasis	250,825 (55.9)	83,660 (18.7)	87,785 (19.6)	14,445 (3.2)	11,265 (2.5)	500 (0.1)	448,480 (100)
	1,726.16	1055.36	856.62	946.61	985.48	1,113.15	1,266.22
Invasive	11,175 (54.3)	4,285 (20.8)	3,890 (18.9)	600 (2.9)	615 (3.0)	†	20,590 (100)
	76.90	54.05	37.94	39.32	53.80		58.12
Noninvasive	218,400 (56.4)	71,345 (18.4)	74,495 (19.3)	12,640 (3.3)	9,665 (2.5)	435 (0.1)	386,980 (100)
	1,503.26	900.01	727.17	828.33	845.51	985.84	1,092.76
Coccidioidomycosis	3,635 (39.1)	2,740 (29.5)	2,220 (23.9)	345 (3.7)	345 (3.7)	†	9,295 (100)
	25.01	34.56	21.65	22.61	30.18		26.24
Cryptococcosis	1,745 (36.0)	1,680 (34.7)	975 (20.1)	325 (6.7)	105 (2.2)	†	4,845 (100)
	12.01	21.19	9.51	21.30	9.19		13.68
Histoplasmosis	2,155 (43.7)	855 (17.3)	1,505 (30.5)	240 (4.9)	170 (3.4)	†	4,930 (100)
	14.83	10.79	14.68	15.73	14.87		13.92
Mucormycosis	515 (36.9)	330 (23.7)	420 (30.1)	65 (4.7)	65 (4.7)	†	1,395 (100)
	3.54	4.16	4.10	4.26	5.69		3.94
Pneumocystosis	3,030 (31.8)	2,705 (28.4)	2,630 (27.6)	860 (9.0)	265 (2.8)	†	9,515 (100)
	20.85	34.12	25.65	56.36	23.18		26.86
Other	79,475 (56.0)	27,460 (19.3)	24,955 (17.6)	5,775 (4.1)	4,125 (2.9)	125 (0.1)	141,915 (100)
	546.99	346.41	243.58	378.45	360.86	283.29)	400.72
Unspecified mycoses	7,900 (54.0)	2,945 (20.1)	2,905 (19.9)	440 (3.0)	430 (2.9)	†	14,630 (100)
	54.38	37.15	28.33	28.83	36.74		41.30
Total hospitalizations‡	344,640 (55.4)	117,915 (19.0)	120,970 (19.5)	21,400 (3.4)	16,225 (2.6)	670 (0.1)	621,840 (100)
	2,372.80	1,487.49	1,180.08	1,402.39	1,419.39	1,563.74	1,755.82
Risk conditions							
Asthma	831,755 (36.6)	637,075 (28.0)	651,185 (28.6)	86,845 (3.8)	63,910 (2.8)	2,590 (0.1)	2,273,360 (100)
	5,732.71	8,036.65	6,353.58	5,691.15	5,590.94	5,869.69	6,418.08
Autoimmune conditions	303,765 (62.8)	51,330 (10.6)	109,345 (22.6)	8,530 (1.8)	10,365 (2.1)	515 (0.1)	483,850 (100)
	2,090.06	647.52	1,066.85	558.99	906.74	1,167.09	1,365.90
Cancer	1,648,155 (57.4)	322,505 (11.2)	753,055 (26.2)	60,895 (2.1)	81.560 (2.8)	3,620 (0.1)	2,869,790 (100)
	11,344.56	4,068.38	7,347.52	3,990.59	7,134.98	8,203.95	8,102.73
COPD	3,176,359 (72.1)	528,225 (12.0)	488,745 (11.1)	89,090 (2.0)	115,770 (2.6)	4,375 (0.1)	4,402,564 (100)
	21,866.17	6,663.52	4,768.66	5,838.27	10,127.72	9,915.01	12,431.08
Cirrhosis	275,610 (58.8)	83,775 (17.9)	79.050 (16.9)	16,230 (3.5)	13,795 (2.9)	12,515 (2.7)	468,950 (100)
	1,897.41	1,056.82	771.29	1,063.59	1,206.81	1,210.68	1,324.16
Cystic fibrosis	5,845 (19.8)	9,990 (33.9)	11,780 (40.0)	675 (0.1)	1,170 (4.0)	†	29,465 (100)
	40.24	126.02	114.91	36.57	102.35		83.19
Diabetes mellitus	5,310,555 (63.4)	1,087,730 (13.0)	1,474,455 (17.6)	269,370 (3.2)	226,500 (2.7)	8,370 (0.1)	8,376,980 (100)
	36,549.87	13,721.63	14,386.19	17,652.44	19,814.54	18968.87	2,3651.08
End-stage renal disease	16,495 (50.5)	4,710 (14.4)	9,090 (27.8)	1,395 (4.3)	940 (2.9)	†	32,665 (100)
	113.55	59.42	88.69	91.42	82.23		92.23
HIV	37,180 (34.1)	42,675 (39.1)	17,655 (16.2)	8,165 (7.5)	3,320 (3.0)	185 (0.2)	109,180 (100)

Infections and risk conditions	Payer type						Total
	Medicare	Medicaid	Private	Self-Pay	Other	Unknown	
	255.93	538.34	172.28	535.07	290.44	419.27	308.29
Immunosuppressive disorders	115,020 (51.3)	33,250 (14.8)	65,155 (29.1)	4,935 (2.2)	5,510 (2.5)	230 (0.1)	224,100 (100)
	791.87	419.45	635.96	323.40	482.02	521.24	632.86
Influenza	142,720 (51.5)	56,915 (20.6)	58,930 (21.3)	11,20 (4.1)	6,820 (2.05)	305 (0.1)	276,950 (100)
	982.06	717.98	574.99	737.89	596.62	691.22	781.86
Myelodysplastic syndrome	67,105 (81.7)	3,020 (3.7)	9,870 (12.0)	615 (0.7)	1,520 (1.8)	1,465 (1.8)	82,170 (100)
	461.93	38.10	96.29	40.30	132.97	141.72	232.01
Neutropenia	79,860 (41.0)	37,430 (19.2)	66,255 (34.0)	5,020 (2.6)	6,085 (3.1)	220 (0.1)	194,870 (100)
	549.63	472.18	646.63	328.97	532.32	498.58	550.22
Osteomyelitis	204,360 (53.0)	77,240 (20.0)	73,795 (19.1)	17,445 (4.5)	12,170 (3.2)	440 (0.1)	385,450 (100)
	1,406.05	974.38	719.68	1,143.21	1,064.65	997.18	1,087.97
Pneumonia	1,613,434 (63.2)	370,160 (14.5)	421,675 (16.5)	78,825 (3.1)	65,615 (2.6)	2795 (0.1)	2,552,502 (100)
	11,101.53	4,669.54	4,114.26	5,165.58	5,740.08	6,334.25	7,205.81
Sepsis	1,686,405 (59.8)	429,545 (15.2)	513,320 (18.2)	112,385 (4.0)	76,250 (2.7)	2,715 (0.1)	2,820,730 (100)
	11,609.30	5,418.68	5,008.44	7,364.85	,6679.21	6,512.98	7,964.60
Transplant history	162,740 (61.0)	27,005 (10.1)	68,255 (25.6)	2,890 (1.1)	5,495 (2.1)	195 (0.1)	266,580 (100)
	1,119.82	340.67	666.02	189.39	480.71	441.93	752.60
Transplant complications	87,340 (60.0)	16,390 (11.3)	37,295 (25.6)	1,760 (1.2)	2,695 (1.9)	†	145,540 (100)
	601.21	106.76	363.95	115.34	235.76		410.96
Tuberculosis	1,300 (35.2)	1,165 (31.6)	780 (21.1)	280 (7.6)	160 (4.3)	†	3,690 (100)
	8.95	14.70	7.61	18.35	14.00		10.42
Total hospitalizations	10,670,000 (59.3)	2,697,710 (15.0)	3,542,045 (19.7)	584,425 (3.2)	494,330 (2.7)	19,590 (0.1)	18,088,100 (100)
	73,439.23	34,031.41	34,559.57	38,298.72	43,244.69	44,396.53	50,843.87

*Data collected from the Healthcare Cost and Utilization Project (HCUP), 2019 (https://www.hcup-us.ahrq.gov/nisoverview.jsp). Values represent no. (%), population rate/100,000 persons. COPD, chronic obstructive pulmonary disease.

†Estimates suppressed in keeping with HCUP protocol.

‡Totals are summed by pathogen without duplication of values included in invasive/non-invasive types.

Appendix Table 4. Age groups of hospitalized patients with diagnosed fungal infections, United States, 2019*

Infections and risk conditions	Age group, y: Pediatric, 0–17 y	Adult, 18–64 y	Senior, >65 y	Total
Fungal Infections				
Aspergillosis	835 (4.8)<br>16.06	8,960 (51.9)<br>53.34	7,455 (43.2)<br>55.55	17,250 (100)<br>48.70
Invasive	280 (3.2)<br>5.39	4,560 (52.3)<br>27.15	3,885 (44.5)<br>28.95	8,725 (100)<br>24.63
Noninvasive	350 (8.2)<br>6.73	2,335 (54.6)<br>13.90	1,590 (37.2)<br>11.85	4,275 (100)<br>12.07
Candidiasis	21,290 (4.7)<br>409.47	204,470 (45.7)<br>1219.08	222,414 (49.6)<br>1657.20	448,480 (100)<br>1266.21
Invasive	850 (4.1)<br>16.35	1,290 (50.0)<br>61.26	9450 (45.9)<br>70.41	20,590 (100)<br>58.13
Noninvasive	18,770 (4.9)<br>361.00	173,825 (44.9)<br>1,034.85	194,380 (50.2)<br>1,448.31	386,980 (100)<br>1,092.58
Coccidioidomycosis	175 (1.9)<br>3.37	6,195 (66.6)<br>36.88	2,925 (31.5)<br>21.9	9,295 (100)<br>26.24
Cryptococcosis	60 (1.2)<br>1.15	3,465 (71.5)<br>20.63	1,320 (27.2)<br>9.84	4,845 (100)<br>13.85
Histoplasmosis	200 (4.1)<br>3.85	2,965 (60.1)<br>17.65	1,765 (35.8)<br>13.15	4,930 (100)<br>13.92
Mucormycosis	100 (7.2)<br>1.91	890 (63.8)<br>5.30	405 (29.0)<br>3.02	1,395 (100)<br>3.94
Pneumocystosis	210 (2.2)<br>4.04	7,130 (74.9)<br>42.45	2,175 (22.9)<br>16.21	9,515 (100)<br>26.86
Other	3,805 (2.7)<br>73,18	68,435 (48.2)<br>407.42	69,675 (49.1)<br>519.14	141,915 (100)<br>400.67
Unspecified mycoses	570 (3.9)<br>10.96	7,315 (50.0)<br>43.55	6,745 (46.1)<br>50.26	14,630 (100)<br>41.31
Total hospitalizations†	26,470 (4.3)<br>509.09	291,510 (46.9)<br>1,735.48	303,855 (48.9)<br>2,264.00	621,840 (100)<br>1,755.67
Risk conditions				
Asthma	226,650 (10.0)<br>4,359.10	1,379,400 (60.7)<br>8,212.12	667,290 (29.4)<br>4,971.93	2,273,360 (100)<br>6,418.47
Autoimmune conditions	11,310 (2.3)<br>217.52	223,540 (46.2)<br>1,330.82	248,995 (51.5)<br>1,855.24	483.850 (100)<br>1,366.07
Cancer	83,590 (2.9)<br>1,607.67	1,107,815 (38.6)<br>6595.27	1,678,345 (58.5)<br>12,505.22	2,869,790 (100)<br>8,102.40
COPD	1,070 (0.0)<br>20.58	1,511,440 (34.3)<br>8,998.21	2,890,010 (65.6)<br>21,533.24	4,402,565 (100)<br>12,429.94
Cirrhosis	1,805 (0.4)<br>34.72	234,240 (49.9)<br>1,394.53	232,905 (49.7)<br>1,735.36	468,950 (100)<br>1,324.01
Cystic fibrosis	7,960 (27.0)<br>153.09	20,480 (69.5)<br>121.93	1,025 (3.5)<br>7.64	29,465 (100)<br>83.19
Diabetes mellitus	40,425 (0.5)<br>777.48	3,526,145 (42.1)<br>20,992.56	4,810,300 (57.4)<br>35,841.18	8,376,980 (100)<br>23,651.08
End-stage renal disease	1600 (4.9)<br>30.77	15,245 (46.7)<br>90.76	15,820 (48.4)<br>117.87	32,665 (100)<br>92.22
HIV	175 (0.2)<br>3.37	96,365 (88.3)<br>573.70	12,640 (11.6)<br>94.18	109,180 (100)<br>308.25
Immunosuppressive disorders	17,185 (7.7)<br>330.51	117,205 (52.3)<br>697.77	89,710 (40.0)<br>668.42	224,100 (100)<br>632.71
Influenza	35,990 (13.0)<br>692.19	109,830 (39.7)<br>653.86	131,125 (47.3)<br>977.0	276,950 (100)<br>781.92
Myelodysplastic syndrome	525 (0.6)<br>10.10	12,155 (14.8)<br>72.36	69,490 (84.6)<br>517.76	82,170 (100)<br>231.99
Neutropenia	27,405 (14.1)<br>527.07	92,230 (47.3)<br>549.08	75,235 (38.6)<br>560.57	194,870 (100)<br>550.18
Osteomyelitis	7,090 (1.8)<br>136.36	229,665 (59.6)<br>1,367 (59.6)	148,695 (38.6)<br>1,107.91	385,450 (100)<br>1,088.26
Pneumonia	125,610 (4.9)<br>2,415.83	901,120 (35.3)<br>5,364.73	1,525,740 (59.8)<br>11,368.17	2,552,505 (100)<br>7,206.59
Sepsis	35,485 (1.3)<br>682.47	1,235,750 (43.8)<br>7,356.92	1,549,450 (54.9)<br>11,544.83	2,820,730 (100)<br>7,963.88
Transplant history	13,420 (5.0)<br>258.30	145,160 (54.5)<br>854.20	107,990 (40.5)<br>804.63	266,580 (100)<br>752.65
Transplant complications	6,475 (4.4)<br>124.53	97,220 (66.8)<br>578.79	41,845 (28.8)<br>311.78	145,540 (100)<br>410.91
Tuberculosis	165 (4.5)	2,290 (62.1)	1,235 (33.5)	3,690 (100)

Infections and risk conditions	Age group, y			Total
	Pediatric, 0–17 y	Adult, 18–64 y	Senior, >65 y	
	3.17	13.63	9.20	10.42
Total hospitalizations	517,940 (2.9)	7,698,275 (42.7)	9,792,240 (54.4)	18,008,695 (100)
	9,961.41	45,830.94	72,961.23	50,844.70

*Data collected from the Healthcare Cost and Utilization Project, 2019 (https://www.hcup-us.ahrq.gov/nisoverview.jsp). Values represent no. (%) population rate/100,000 persons. COPD, chronic obstructive pulmonary disease.

†Totals are summed by pathogen without duplication of values included in invasive or noninvasive types.

Appendix Table 5. Urban or rural status of hospitalized patients with diagnosed fungal infections, United States, 2019*

Infections and risk conditions	Classification, population						Urban/rural population rate (95% CI)
	Central metro counties, >1 million	Fringe metro counties, >1 million	Urban, 250,000–999,999	Urban, 50,000–249,999	Rural,10,000–49,999	Rural, <10,000	
Fungal infections							
Aspergillosis	5,860 (33.97) 56.17	4,030 (23.36) 47.15	3,435 (19.91) 46.38	1,455 (8.43) 44.56	1,280 (7.42) 39.89	1,050 (6.09) 44.57	1.19 (1.14–1.24)
Invasive	2,920 (33.47) 27.99	2,070 (23.72) 24.22	1,790 (20.52) 24.17	720 (8.25) 22.05	615 (7.05) 19.16	530 (6.07) 22.50	1.23 (1.16–1.31)
Noninvasive	1,525 (35.67) 14.62	1,025 (23.98) 11.99	875 (20.47) 11.81	420 (9.82) 12.86	130 (3.04) 4.05	170 (3.98) 7.22	1.80 (1.63–2.00)
Candidiasis	122,470 (27.31) 1,173.98	105,925 (23.62) 1,239.41	97,300 (21.70) 1,313.66	42,550 (9.49) 1,302.99	44,770 (9.98) 1,385.09	32,915 (7.34) 1,397.22	0.89 (0.88–0.90)
Invasive	6,135 (29.80) 58.81	4,495 (21.83) 52.60	4,280 (20.79) 57.78	1,790 (8.69) 54.81	2,220 (10.78) 69.18	1,525 (7.41) 64.74	0.84 (0.81–0.87)
Noninvasive	104,920 (27.11) 1,005.75	91,425 (23.63) 1,069.75	84,110 (21.73) 1,135.58	37,105 (9.59) 1,136.25	38,990 (10.08) 1,214.97	28,265 (7.30) 1,199.83	0.89 (0.88–0.89)
Coccidioidomycosis	3,910 (42.07) 37.48	915 (9.84) 10.71	3,275 (35.23) 44.22	575 (6.19) 17.61	300 (3.23) 9.35	175 (1.88) 7.43	3.43 (3.13–3.76)
Cryptococcosis	1,675 (34.57) 16.06	880 (18.16) 10.30	915 (18.89) 12.35	455 (9.39) 13.93	365 (7.53) 11.37	395 (8.15) 16.77	0.97 (0.89–1.05)
Histoplasmosis	1,275 (25.86) 12.22	1,015 (20.59) 11.88	910 (18.46) 12.29	545 (11.05) 16.69	625 (12.68) 19.48	520 (10.55) 22.07	0.61 (0.58–0.66)
Mucormycosis	465 (33.33) 4.46	375 (26.88) 4.39	270 (19.35) 3.65	90 (6.45) 2.76	115 (8.24) 3.58	70 (5.02) 2.97	1.22 (1.05–1.43)
Pneumocystosis	4,160 (43.72) 39.88	2,210 (23.23) 25.86	1,545 (16.24) 20.86	600 (6.31) 18.37	465 (4.89) 14.49	375 (3.94) 15.92	1.90 (1.77–2.04)
Other	48,990 (34.27) 469.61	34,035 (23.81) 398.24	28,550 (19.97) 385.46	11,010 (7.70) 337.15	10,860 (7.60) 338.41	7,505 (5.25) 318.58	1.27 (1.25–1.28)
Unspecified mycoses	4,450 (30.42) 42.66	3,660 (25.02) 42.83	2,785 (19.04) 37.60	1,315 (8.99) 40.27	1,260 (8.61) 39.26	1,040 (7.11) 44.15	1.00 (0.95–1.04)
Total hospitalizations	182,075 (29.28) 1,745.35	147,225 (23.68) 1,722.65	131,400 (21.13) 1,774.04	56,085 (9.02) 1,717.47	57,800 (9.29) 1,801.12	42,390 (6.82) 1,799.43	0.97 (0.96–0.98)
Risk conditions							
Asthma	772,790 (33.99) 7,407.87	564,525 (24.8) 6,605.41	463,620 (20.39) 6,259.38	190,685 (8.39) 5,839.26	163,355 (7.19) 5,090.33	105,625 (4.65) 4,483.473	1.39 (1.38–1.40)
Autoimmune conditions	126,655 (26.18) 1,213.56	130,320 (26.93) 1,524.85	103,655 (21.42) 1,399.46	48,130 (9.95) 1,473.86	43,665 (9.02) 1,360.65	30,120 (6.23) 1,278.57	1.04 (1.03–1.05)
Cancer	838,145 (29.21) 8,034.35	735,175 (25.62) 8,602.16	578,815 (20.17) 7,814.64	257,855 (8.99) 7,896.18	254,220 (8.86) 7,921.78	194,620 (6.78) 8,261.52	1.01 (1.01–1.01)
COPD	1,045,360 (23.74) 10,020.69	990,835 (22.51) 11,593.59	929,299 (21.11) 12,546.56	487,735 (11.08) 14,935.70	520,706 (11.83) 16,225.79	406,179 (9.23) 17,242.09	0.70 (0.70–0.70)
Cirrhosis	139,245 (29.69) 1,335.14	101,305 (21.54) 1,182.19	102,135 (21.78) 1,378.94	43,840 (9.35) 1,342.49	45,680 (9.74) 1,423.44	33,115 (7.06) 1,405.71	0.92 (0.91–0.93)
Cystic fibrosis	7,185 (24.38) 68.91	7,545 (25.61) 88.28	6,615 (22.45) 89.31	2,920 (9.67) 89.42	2,850 (9.67) 89.81	2,205 (7.48) 93.60	0.90 (0.87–0.93)
Diabetes mellitus	2,456,535 (29.09) 23,356.31	1,935,565 (23.11) 22,647.71	1,740,272 (20.77) 23,495.62	791,550 (9.45) 24,239.29	816,397 (9.67) 25,439.85	619,050 (7.46) 26,338.80	0.90 (0.90–0.90)
End-stage renal disease	11,000 (33.68) 105.45	8,235 (25.21) 96.36	6,345 (19.42) 85.66	2,680 (8.20) 82.07	2,325 (7.11) 72.45	1,900 (5.82) 80.65	1.26 (1.22–1.30)

Infections and risk conditions	Classification, population						Urban/rural population rate (95% CI)
	Central metro counties, >1 million	Fringe metro counties, >1 million	Urban, 250,000–999,999	Urban, 50,000–249,999	Rural,10,000–49,999	Rural, <10,000	
HIV	57,095 (52.29) 547.56	18,600 (17.04) 217.64	18,435 (16.88) 248.89	5,735 (5.25) 175.62	3,925 (3.59) 122.31	2,925 (2.26) 104.64	2.93 (2.86–3.01)
Immunosuppressive disorders	67,480 (30.11) 647.14	59,315 (26.47) 694.03	46,070 (20.56) 622.00	19,920 (8.89) 610.00	17,450 (7.79) 543.76	13,105 (5.85) 556.30	1.18 (1.17–1.20)
Influenza	80,280 (28.99) 769.68	64,670 (23.35) 756.69	59,000 (21.30) 796.56	25,705 (9.28) 787.15	26,305 (9.50) 819.70	19,665 (7.1) 834.76	0.94 (0.93–0.95)
Myelodysplastic syndrome	22,395 (27.31) 214.68	23,295 (28.31) 272.57	16,695 (20.36) 225.40	7,580 (9.24) 232.12	6,860 (8.37) 213.77	5,180 (6.37) 219.89	1.09 (1.07–1.11)
Neutropenia	59,330 (30.66) 568.73	50,240 (25.96) 587.85	39,895 (20.62) 538.63	16,665 (8.61) 510.33	15,600 (8.06) 486.11	11,765 (6.08) 499.42	1.14 (1.13–1.15)
Osteomyelitis	117,340 (30.73) 1,124.81	88,070 (23.07) 1,030.49	80,165 (21.00) 1,082.32	33,715 (8.83) 1,032.44	36,005 (9.43) 1,121.96	26,530 (6.95) 1,121.96	0.96 (0.95–0.97)
Pneumonia	666,010 (26.09) 6,384.29	581,085 (22.77) 6,799.18	524,365 (20.54) 7,079.51	257,570 (10.09) 7,887.45	282,570 (11.07) 8,801.80	228,369 (8.95) 9,694.15	0.75 (0.74–0.75)
Sepsis	839,780 (29.77) 8,050.02	653,275 (23.16) 7,643.86	601,720 (21.33) 78,123.88	263,450 (9.34) 8,067.51	260,880 (9.25) 8,129.32	183,180 (6.49) 7,775.90	1.00 (0.99–1.00)
Transplant history	83,315 (31.25) 798.55	70,600 (26.48) 826.08	52,855 (19.83) 713.60	22,650 (8.50) 693.60	21,780 (8.17) 678.69	14,405 (5.40) 611.48	1.19 (1.18–1.20)
Transplant complications	51,475 (35.37) 493.46	38,115 (26.19) 445.98	27,275 (18.74) 368.24	10,575 (7.27) 323.83	10,355 (7.11) 322.67	7,215 (4.96) 306.27	1.36 (1.34–1.38)
Tuberculosis	1,700 (46.07) 16.30	810 (21.95) 9.48	565 (15.31) 7.63	245 (6.64) 7.50	165 (4.47) 5.14	140 (3.79) 3.79	1.95 (1.82–2.30)
Total hospitalizations	5,163,780 (28.67) 49,499.33	4,283,634 (23.79) 50,122.06	3,750,863 (20.83) 50,640.80	1,706,020 (9.47) 52,242.70	1,718,793 (9.54) 53,559.54	1,292,533 (7.18) 54,867.36	0.93 (0.93–0.93)

*Data collected from the Healthcare Cost and Utilization Project, 2019 (https://www.hcup-us.ahrq.gov/nisoverview.jsp). Values represent no. (%), population rate/100,000 persons. COPD, chronic obstructive pulmonary disease.

Highlighting indicates the following rate ratios: <0.75 | 1.25–1.49 | 1.50–1.99 | 2.00–2.99 | >3.00
